# Catalytically Propelled Micro‐ and Nanoswimmers

**DOI:** 10.1002/smsc.202300076

**Published:** 2023-09-24

**Authors:** Bumjin Jang, Min Ye, Ayoung Hong, Xiaopu Wang, Xianghong Liu, Dohyeok Bae, Josep Puigmartí Luis, Salvador Pané

**Affiliations:** ^1^ Department of Robotics Hanyang University ERICA Campus Ansan 15588 Republic of Korea; ^2^ Shenzhen Institute of Artificial Intelligence and Robotics for Society The Chinese University of Hong Kong Shenzhen 518129 China; ^3^ Department of Mechanical Engineering Chonnam National University Gwangju 61186 Republic of Korea; ^4^ Departament de Ciencia dels Materials i Quimica Fisica Institut de Quimica Teorica i Computacional 08028 Barcelona Spain; ^5^ Institucio Catalana de Recerca i Estudis Avancats (ICREA) Pg. Lluis Companys 23 08010 Barcelona Spain; ^6^ Multi-Scale Robotics Lab Institute of Robotics and Intelligent Systems, ETH Zurich 8092 Zurich Switzerland

**Keywords:** bubble-recoil process, chemical propulsion, micro- and nanoswimmers, self-diffusiophoresis, self-electrophoresis

## Abstract

The last decade has seen a surge of interest in the field of catalytically propelled micro‐ and nanoswimmers for their potential use in biomedical applications, such as biosensing, biopsy, targeted drug delivery, and on‐the‐fly chemistry. However, to fully utilize these devices, precise control over their motion is essential. Therefore, it is important to thoroughly understand their locomotion mechanisms. Herein, the currently accepted mechanisms for propulsion are discussed, which are self‐electrophoresis, self‐diffusiophoresis, and bubble recoil. Additionally, the concept of using multilocomotive mechanisms as a solution to achieve fully autonomous navigation is explored. Moreover, recent advances in the design of these devices are explored.

## Graphical abstract

One of the challenges in the field of catalytic micro‐ and nanoswimmers is their precise motion control. It has been established that self‐electrophoresis, self‐diffusiophoresis, and bubble recoil are motion mechanisms for catalytic micro‐ and nanoswimmers. Recently, the concept of a multilocomotive mechanism has gained significant attention as a potential solution to address this challenge and enable fully autonomous navigation.

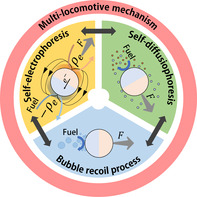

## 1 Introduction

Over the past decades, there has been a growing interest in micro‐ and nanoswimmers due to the demand for small‐scale agents capable of precise control and intelligent operations in complex and confined areas. A major challenge in developing such small‐scale swimmers is finding a suitable power source, as it is impractical to incorporate internal batteries within their limited volume. To overcome this challenge, researchers have explored the use of external energy sources such as magnetic, electric, acoustic, and optical energy.^[^ 1, 2, 3, 4 ^]^ However, these methods have limitations, including insufficient penetration depth, high costs, and the need for an additional monitoring system for feedback control. Taking inspiration from nature, particularly microorganisms, researchers have employed chemical reactions as a potential solution to address the power challenge. Microorganisms derive their energy from dynamic in situ chemical processes, obtaining nutrients from the surrounding liquid media.^[^ ^5^ ^]^ Building upon this concept, researchers have sought to replicate these natural powering methods using chemical reactions.

One subcategory of chemically driven small‐scale swimmers is catalytically propelled small‐scale swimmers, which can move in fluids by catalyzing chemical reactions with species present in their surrounding fluid environment.^[^ ^6^ ^]^ Advances in materials and fabrication techniques, particularly in nanotechnology, have enabled the miniaturization of these devices to the nanoscale. A significant body of research has focused on designing these machines, often employing Pt (platinum) building blocks as catalytic components and H_2_O_2_ (hydrogen peroxide) as the fuel. However, due to their applications in biomedical fields,^[^ 7, 8, 9 ^]^ recent efforts have been directed toward substituting Pt with other more sustainable and biocompatible materials, such as MnO_2_^[^ 10, 11, 12 ^]^ or enzymes,^[^ 7, 8, 13, 14 ^]^ and finding alternative fuels to replace toxic H_2_O_2_.^[^ 15, 16, 17, 18 ^]^ Examples of alternative biological fuels include acidic and basic aqueous solutions, as well as solutions containing iodine, glucose, and urea.^[^ ^8^ ^]^ In addition to addressing the cytotoxicity of the fuel solutions, another challenge in motion controllability needs to be addressed to realize full autonomous navigation for biomedical applications. One strategy to achieve directional change in motion is to tune the propulsion mechanism of the swimmer by sensing environmental changes, similar to how microorganisms exhibit autonomous directional change through positive and negative chemotaxis.^[^ 19, 20 ^]^ Therefore, gaining a comprehensive understanding of the propulsion mechanisms employed by catalytically propelled swimmers is crucial for the advancement of this field.

Two conditions are required for generating propulsion in small‐scale structures. First, swimmers must be designed such that, a part or a region of their outermost surface is catalytically active and can react with species in the surrounding solution to generate byproducts. For example, a catalytic layer decomposes a compound present in the swimming media.^[^ ^21^ ^]^ Second, the swimmers should display certain asymmetry, which can be either in material composition (e.g., a sphere half‐coated with a reactive material),^[^ 22, 23, 24 ^]^ geometry(e.g., a boomerang‐like morphology),^[^ 25, 26 ^]^ or surface topology (e.g., surface roughness).^[^ 27, 28, 29 ^]^ The asymmetry allows a chemical gradient to develop around the swimmers, which is ultimately responsible for their motion. Nevertheless, since the pioneering work from Whitesides’ team,^[^ ^30^ ^]^ sufficient information on how the formed chemical gradient physiochemically develops its final locomotion is still lacking and, therefore, needs to be resolved. In detail, the self‐propelled millimeter‐scale polydimethylsiloxane (PDMS) ships with catalytic Pt‐coated keels, engineered by Whitesides’ team moved autonomously in a solution of hydrogen peroxide (H_2_O_2_).^[^ ^30^ ^]^ The decomposition of H_2_O_2_ into water and oxygen at the keel surface generates propulsion as oxygen bubbles impart momentum to the ship. Despite unleashing a wealth of both fundamental and applied research on chemical propulsion at small scales by the discovery, the bubble imparting propulsion mechanism (or bubble recoil process) for the millimeter‐scale ship, could not universally explain the motion of several small‐scale swimmer designs. Instead, other propulsion mechanisms, such as self‐electrophoresis or self‐diffusiophoresis had to be considered, depending on the swimmer's design parameters, such as geometry, size, and composition. Such controversy is probably a result of insufficient experimental data, such as the quantitative determination of specific chemical species around the swimmers, to establish concrete propulsion mechanisms. For example, Fournier‐Bidoz et al. reported that nanobubbles might be responsible for the propulsive force of Au/Ni bimetallic nanorods in H_2_O_2_.^[^ ^31^ ^]^ ${\text{SiO}}_{2}/\text{Pt}$ Janus microspheres were also suggested in following this mechanism for their propulsion in H_2_O_2_ solution.^[^ ^32^ ^]^ However, as nanosized bubbles cannot be characterized in terms of size and quantity, it is challenging to verify if the bubbles indeed create sufficient thrust to propel these structures.

In this article, we review the widely accepted propulsion mechanisms of catalytically propelled micro‐ and nanostructures, taking factors into account that could influence the swimmers’ motion dynamics. In particular, theories on three propulsion mechanisms, including self‐electrophoresis, self‐diffusiophoresis, and bubble recoil, are thoroughly discussed in Sections 2.1, 2.2, and 2.3. Recent findings on the multipropulsive mechanisms are also discussed in Section 2.4. In addition to the theories behind each propulsion mechanism, we also review up‐to‐date designs of catalytically propelled swimmers.

## 2 Catalytic Micro‐ and Nanoswimmers

### 2.1 Self‐Electrophoretic Swimmers

#### 2.1.1 Fundamentals of Self‐Electrophoretic Locomotion Mechanism

**Figure 1:**
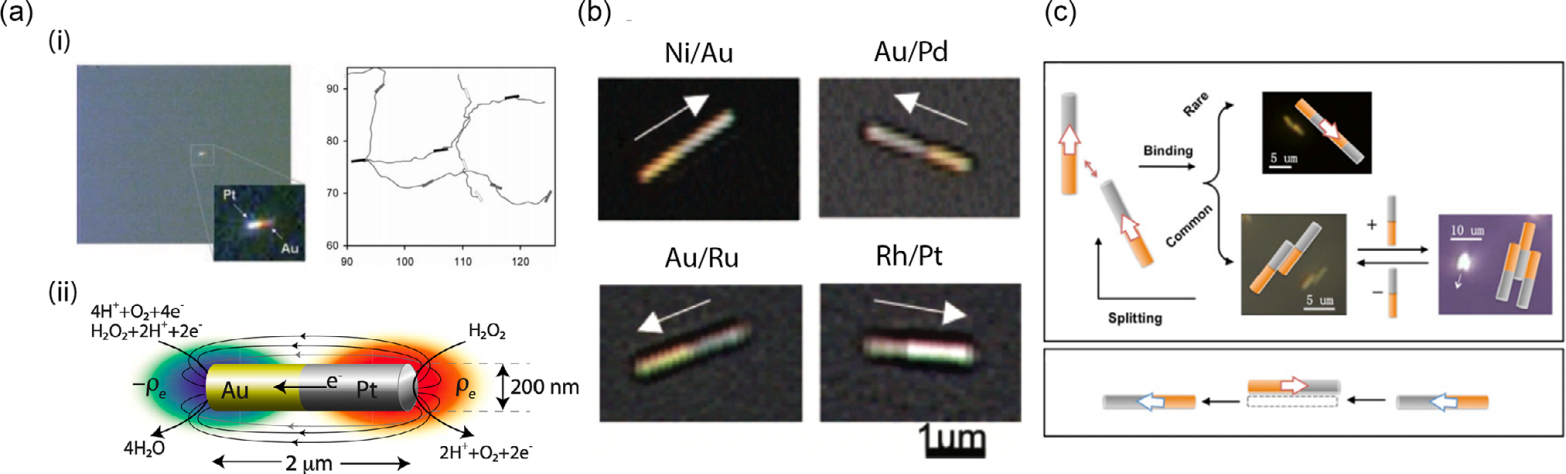
Self‐electrophoretic mechanism of bimetallic nanoswimmers. a) i) An optical image (500×) of a Au/Pt bimetallic nanoswimmers (left), and a trajectory plot of three Au/Pt nanoswimmers in 2.5% H_2_O_2_ over 5 s (right). Adapted with permission.^[^ ^6^ ^]^ Copyright 2004, American Chemical Society. Adapted with permission.^[^ ^34^ ^]^ Copyright 2005, Wiley‐VCH. ii) A scheme of the self‐electrophoretic mechanism of bimetallic nanoswimmers. Adapted with permission.^[^ ^35^ ^]^ Copyright 2010, American Physical Society. b) A prediction of the propulsive direction of nanoswimmers with various material combinations, based on a Tafel analysis. Adapted with permission.^[^ ^39^ ^]^ Copyright 2006, American Chemical Society. c) A cluster of the bimetallic nanoswimmers, formed by a self‐generated dipole–dipole interaction. Adapted with permission.^[^ ^42^ ^]^ Copyright 2006, National Academy of Sciences, USA.

The first generation of nanoscale catalytic machines was reported by Paxton et al. in 2004.^[^ ^6^ ^]^ They observed that the bimetallic nanorods moved with a certain net displacement (**Figure** **1**a(i)). However, they failed to predict the motion direction of the nanoswimmers with the bubble recoil model proposed by Ismagilove et al., which claims that a chemically reactive millimeter ship moves in the direction of a noncatalytic component end against the releasing force of the O_2_ bubbles at the catalytic component end.^[^ ^30^ ^]^ To explain the unexpected behavior of the nanoswimmers, a model based on the interfacial tension gradient between the solution and the solid body of nanoswimmers was proposed.^[^ ^6^ ^]^ The tension is lower on the Pt surface owing to the O_2_ generated by H_2_O_2_ decomposition, whereas there is no tension change in the Au segment. Fluid moves from the regions exhibiting lower surface tension to those exhibiting a higher surface tension, leading to the motion of the nanoswimmers in a direction opposite to the fluid flow. The second model, by Dhar et al. in 2006, suggests that the friction gradient formed by the asymmetrical evolution of O_2_ can be attributed to the translational motion of the nanoswimmers.^[^ ^33^ ^]^ Friction gradually decreases in the direction of the platinum end, where O_2_ is continuously generated by the H_2_O_2_ decomposition. Consequently, the nanoswimmers move in the direction where friction is minimal. These two models successfully explain the motion of Au/Pt nanoswimmers but fail to explain the mechanism for other metallic nanoswimmers consisting of different metallic layers. The third model is the self‐electrophoretic model based on redox electrochemistry, which is currently the accepted propulsion mechanism for catalytic bimetallic systems.

According to the self‐electrophoretic model proposed by Paxton et al.^[^ ^34^ ^]^ and further detailed by Moran et al.,^[^ 35, 36 ^]^ a bimetallic nanoswimmer forms a short‐circuited galvanic cell in the presence of a fuel solution (H_2_O_2_), with Pt and Au segments as the cathode and the anode, respectively (Figure 1a(ii)). Protons, oxygen molecules, and electrons are generated by decomposing H_2_O_2_ at the anode (Pt) through oxidation. The generated protons travel to the cathode (Au) by convection, diffusion, and migration, where they subsequently reduce to water by reacting with electrons, oxygen, and H_2_O_2_ molecules. The proton transport can be mathematically described by combining the Nernst–Planck equation and the continuity equation

(1) $$\nabla \cdot J_{i}=0=u\cdot \nabla c_{i}-D_{i}\nabla^{2}c_{i}-\frac{z_{i}FD_{i}\nabla \cdot (c_{i}\nabla \phi )}{RT}$$

where, *J* is the flux, *u* is the fluid velocity, *c* is the concentration, *R* is the gas constant, *T* is the temperature, *ϕ* is the electrostatic potential, and *F* is Faraday constant. *z* and *D* are the valence electron and the diffusion constant of the protons, respectively. The subscript *i* denotes the *i*‐th ion species, which are $H^{+}$ and ${\text{HCO}}_{3}^{-}$ for the redox reaction of Au/Pt nanorod in H_2_O_2_. The terms on the right side of Equation (1) represent the diffusion, convection, and electromigration of protons, from left to right. As a consequence of these redox reactions, protons are asymmetrically distributed around the nanorod and develop an asymmetric electric field. This can be expressed by the Poisson equation that couples the space charge density ($\rho_{e}$) with the electrostatic potential (*ϕ*), as follows

(2) $$-\varepsilon \nabla^{2}\phi =\rho_{e}=F(z_{+}c_{+}+z_{-}c_{-})$$

where, *ε* is the permittivity of water, and $\rho_{e}$ is the space charge density, which is the sum of $H^{+}$ and ${\text{HCO}}_{3}^{-}$ ($z_{+}$ and $z_{-}$, respectively). Note that the electrostatic potential (*ϕ*) is often treated as the zeta potential (*ζ*) of the swimmer, which is a quantitative value used to describe the effective potential in the electrical double layer (EDL) for the phoretic model. The self‐generated potential causes an electroosmotic flow by proton migration in the EDL of the swimmer. The flow around the swimmer can be described by incorporating an electroosmotic body force in the Stokes' equation (Equations (3) and (4)). To solve this equation, slip‐velocity conditions are set for the surface boundary, and the fluid is considered incompressible.

(3) ∇⋅u=0

(4) $$\rho (u\cdot \nabla u)=-\nabla p+\eta \nabla^{2}u-\rho_{e}\nabla \phi$$

where *ρ* is the density of water, *p* is the pressure, *η* is the dynamic viscosity of water, and $\rho_{e}\nabla \phi$ is the body force. The velocity of the swimmer can be deduced using the Helmhotz–Smoluchowski equation (Equation (5)). This equation is widely used to estimate the speed of small colloidal particles that show phoretic motion under a uniform electric field regardless of their shape.

(5) $$U_{\text{swimmer}}=\varepsilon \frac{\zeta_{\varepsilon }E}{\eta }$$

where *ζ* is the zeta potential of the swimmer, *η* is the dynamic viscosity of water, *ε* is the solution permittivity, and *E* is the self‐generated electric field (E=−∇ϕ) from Equation (2). Wang et al. adopted a method proposed by Solomentsev et al. to precisely estimate the speed of the rod‐shaped swimmer, generated by the nonuniform field distribution around the swimmer.^[^ 37, 38 ^]^ The equation converts the speed of osmosis flow at the surface with a slip boundary condition, taking the shape factor into account. The speed obtained using the Solomentsev method shows a similar value to the one obtained using the conventional Helmhotz–Smoluchowski equation (Equation (6)).

(6) $$U_{\text{swimmer}}=-\frac{<\alpha U_{\text{slip}}>}{<\alpha >}$$

where

(7) $$\alpha \;=\;\frac{1-2\ln(2\varepsilon {\left(1-s^{2}\right)}^{1/2})}{8(1-\ln\varepsilon {)}^{2}}\;\;\text{and}\;\;\varepsilon \;=\;\frac{b_{0}}{L/2}$$

and where $U_{\text{swimmer}}$ is the speed of the swimmer, $U_{\text{slip}}$ is the slip velocity along the surface of the swimmer, and *L* and $b_{0}$ are the length and the radius of the swimmer, respectively. The relative speed of bimetallic nanoswimmers with different materials, such as metals,^[^ ^39^ ^]^ and alloys^[^ ^40^ ^]^ in different fuel solutions,^[^ ^41^ ^]^ can be predicted by Tafel experiments, which compare the potential differences between an anode and a cathode in a swimmer system. In addition to the speed of the swimmer, the measured electrochemical potential difference can predict the motion direction of the swimmer, demonstrating that the more anodic segment present in a bimetallic system of the swimmer leads the less anodic one (Figure 1b). Another interesting discovery is that the electrochemical potential difference forms a cluster among neighboring swimmers and/or with surrounding charged objects. For instance, an attractive force (van der Waals force or electrostatic force) is developed when the segment of swimmers with opposite dipoles are close to each other (Figure 1c).^[^ ^42^ ^]^

#### 2.1.2 Designs of Self‐Electrophoretic Swimmers

**Figure 2:**
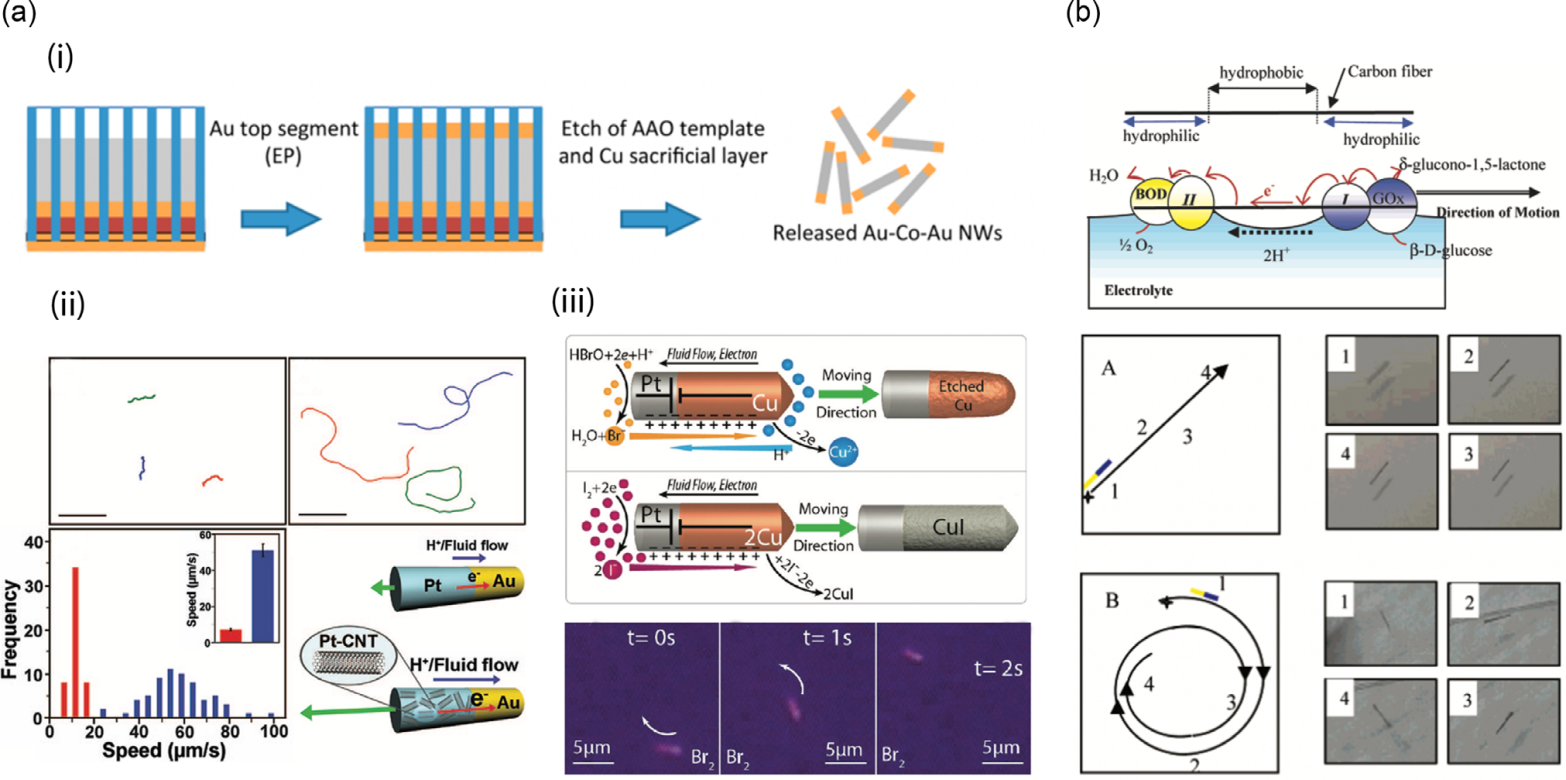
Structural examples of bimetallic nanoswimmers with a rod shape and their electrophoretic mechanism. a) Nanosized electrophoretic swimmers with a rod configuration. i) A brief illustration of the synthesis of multisegmented nanowires. Adapted with permission.^[^ ^43^ ^]^ Copyright 2014, American Chemical Society. ii,iii) Configuration and motion of Au/ Pt‐CNT (ii) and Pt/Cu (iii) nanoswimmers. ii) Adapted with permission.^[^ ^44^ ^]^ Copyright 2008, American Chemical Society. iii) Adapted with permission.^[^ ^41^ ^]^ Copyright 2011, American Chemical Society. b) Microsized electrophoretic swimmers with a rod configuration. Gx and BOD are coated at each end of the carbon rod. Adapted with permission.^[^ ^46^ ^]^ Copyright 2005, American Chemical Society.

The self‐electrophoretic mechanism is mostly observed in structures such as bimetallic nanorods and micro‐ and nanospheres in the presence of a fuel solution. The bimetallic swimmers with rod shapes can be synthesized by carrying out sequential electrodeposition of two different materials into a porous anodic aluminum oxide (AAO) or a track‐etched membrane, as shown in **Figure** **2**a(i).^[^ ^43^ ^]^ Subsequently, the AAO membrane is etched away in a NaOH solution to release the swimmers. Applying a straightforward and cost‐effective method, various bimetallic segments can be obtained by simply changing the electrolytes during the deposition process. In addition to the Au/Pt segmented structure, a complex compound of Pt‐CNT carbon nanotubes can be synthesized by simply adding CNTs to Pt electrolytes during the plating of the Pt segment. The synthesized Au/Pt‐CNT nanorods, as reported in Laocharoensuk et al., present a largely enhanced speed of 51.0 μm s^−1^ compared to the conventional Au/Pt nanorods (7.2 μm s^−1^) in the presence of 15 wt% H_2_O_2_ solution (Figure 2a(ii)).^[^ ^41^ ^]^ A Tafel analysis reveals that the measured electrode potential of Au/Pt‐CNT is higher than that of Pt/Au, indicating an increased redox chemical reaction in the fuel solution. The author also claims that the speed of the swimmer exceeds 200 μm s^−1^ with the hydrazine additive in H_2_O_2_, which further improves the redox reaction. The Cu/Pt nanorod presented in Liu et al. is another example of a bimetallic type nanorod (Figure 2a(iii)).^[^ ^44^ ^]^ Interestingly, with a self‐electrophoretic mechanism, the bimetallic nanorod moves in both aqueous Br_2_ and I_2_. One of the key findings is that the Cu segment is dissolved in the Br_2_ fuel solution or is converted to CuI in the I_2_ fuel solution by the reduction and oxidation process, respectively; hence, the Cu segment has a short lifetime. Unfortunately, in this report, the influence of the dynamics of the byproducts ${\text{Cu}}_{2}^{+}$ and CuI on the self‐electrophoretic mechanism could not be clarified. Using the electrodeposition technique, alloys and multimetallic segments can also be obtained to achieve multifunctionalities for the swimmers, such as a magnetic guidance and molecular bindings.^[^ 9, 13, 34, 45 ^]^ Such functionalities do not affect the motion dynamics of the swimmers and are therefore not discussed these cases in detail in this article. Although the self‐electrophoretic mechanism is mainly observed in nanoswimmers, it has also been found in a few self‐electrophoretic microswimmers (Figure 2b). A carbon fiber with a length of 8 mm and a diameter of 7 μm underwent an O_2_ plasma treatment to acquire a hydrophilic surface at its two ends while retaining hydrophobic at center.^[^ ^46^ ^]^ The hydrophilic surface can be used to electrostatically bind glucose oxidase (GOx) and a redox polymer at one end, while the other end is functionalized with bilirubin oxidase (BOD) and another redox polymer. In a biofuel composed of a pH 7 buffer solution and 10 mm glucose additive, self‐electrophoresis occurs around the microswimmer as the anode and cathode reactions occur at the GOx and BOD sites, respectively, thereby creating a net motion at the interface of air and the solution. Their propulsion speed has been improved and various fuel solutions are used to enable their precise manipulation in complex environments, such as physiological liquid environments.

**Figure 3:**
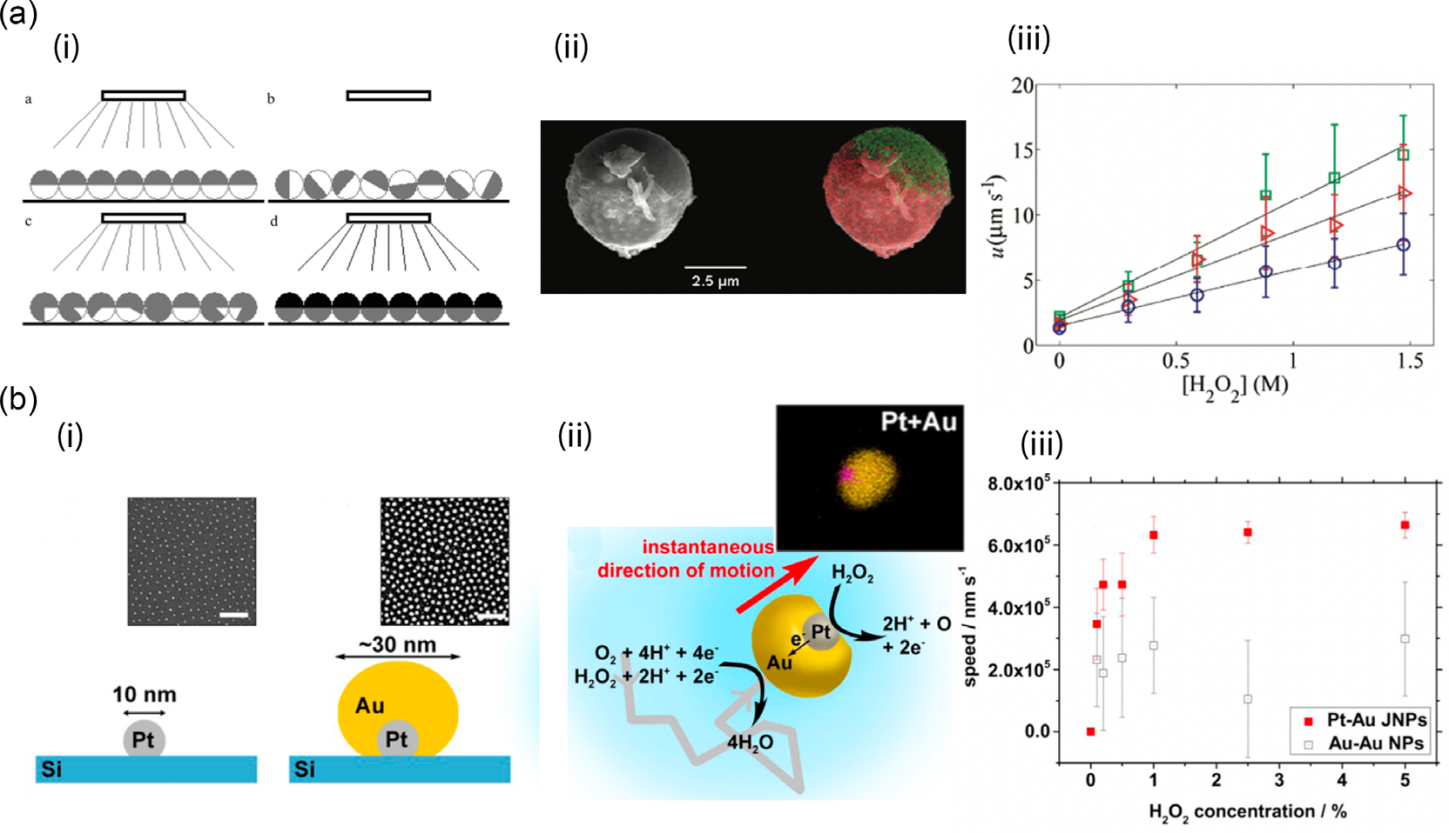
a,b) Bimetallic microswimmers (a) and nanoswimmers (b) with spherical shapes propelled by the electrophoretic mechanism. a) i) A scheme of the fabrication process of the Au/Pt microswimmer, ii) SEM images of the bimetallic microswimmers, and iii) speeds of the swimmers as a function of H_2_O_2_ concentration. Adapted with permission.^[^ ^47^ ^]^ Copyright 2010, American Chemical Society. b) i) Fabrication scheme of bimetallic Au/Pt nanoswimmers using a glancing angle deposition technique, ii) a TEM image of Au/Pt nanoswimmers and the scheme of the swimmer's ballistic motion, and iii) swimmer speeds as a function of H_2_O_2_ concentration. Adapted with permission.^[^ ^22^ ^]^ Copyright 2014, American Chemical Society.

Another bimetallic configuration for the redox‐based reactive swimmers is a spherical structure. Wheat et al. successfully synthesized Au/Pt Janus microstructures by sequentially sputtering Au and Pt layers onto the surface of fluorescent polystyrene (PS) beads.^[^ ^47^ ^]^ As depicted in **Figure** **3**a(i), first a monolayer of PS beads on a glass substrate was achieved by dispersing PS beads mixed with a solvent, followed by solvent evaporation at RT. Au sputtering was conducted several times (7–8) on the top surface of the PS monolayer: the Au sputtering was followed by the processes of redispersion of the beads, so that the beads would be entirely covered with an Au layer. Finally, Au/Pt Janus microspheres were then formed after sputtering a Pt layer on top of a hemisphere of Au‐coated PS beads (Figure 3a(ii)). The fluorescent property of PS beads was used to verify the coverage of the Au layer after each sputtering process. Like bimetallic nanoswimmers, the synthesized Janus microswimmer also displayed a net motion in the presence of H_2_O_2_ and moved with the same motile mechanism. It is worth noting that, in this system, the swimmer exhibits more directional motion compared with the previously reported nanorod types due to the decrease in the rotational Brownian motion as the size of the diameter of the swimmer increases from 200 nm to 3 μm. In addition, as the diameter increases the speed of the swimmer decreases due to the increase in the viscous force on the longer characteristic length (Figure 3a(iii)).

Lee et al. demonstrated that Au/Pt Janus nanostructures with an overall size of 30 nm can be prepared by depositing an Au layer using the glancing angle deposition method on well‐patterned Pt nanoparticles fabricated by a block copolymer micelle lithography technique (Figure 3b(i)).^[^ ^22^ ^]^ The motion of the Au/Pt Janus spheres is dominated by collision with the surrounding liquid molecules due to their nanoscale size, while the propulsion method of the swimmer is attributed to self‐electrophoresis (Figure 3b(ii)), resulting in a burst of linear ballistic motion (Figure 3b(iii)).

### 2.2 Self‐Diffusiophoretic Swimmers

#### 2.2.1 Fundamentals of Self‐Diffusiophoretic Locomotion Mechanism

**Figure 4:**
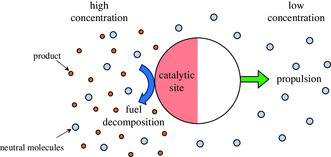
Scheme of the spherical catalytic microswimmer driven by self‐diffusiophoresis.

Self‐diffusiophoresis is a mechanism that enables catalytic micro‐ and nanoswimmers to move through a solution by utilizing a solute concentration gradient created by the decomposition of a fuel at a catalytic site. The concentration gradient drives the motion of the swimmer as the solute diffuses from an area of high concentration to an area of low concentration (**Figure** **4**).

The term diffusiophoresis was originally introduced by Deryagin et al. in 1961 to describe the relative motion between two phases that is induced by a gradient in solute concentration.^[^ ^48^ ^]^ Any molecule dissolved in a solution can move from an area of higher concentration to that of lower concentration. However, when a rigid particle is placed in the solution, it disturbs the solute concentration, which produces stresses near the particle surface. These stresses cause the motion of the fluid. The exact interaction between the particle and solute has not yet to be experimentally validated, however, the excluded volume effect is commonly considered in modeling the interaction potentials. In 1982, Anderson et al. theoretically analyzed the motion of a rigid spherical particle placed in a fluid with a nonuniform solute concentration.^[^ ^49^ ^]^ The disturbed concentration field −∇ψ results in the body force $f_{b}=-c\nabla \psi$, where *c* is the solute concentration. The fluid Equations (3) and (4) can be rewritten by incorporating the diffusiophoretic force as

(8) ∇⋅u=0

(9) $$\eta \nabla^{2}u=\nabla p-c\nabla \psi$$

where *η* is the fluid viscosity and *p* is the pressure. The slip velocity between the particle and the solution is obtained as

(10) $$U=\frac{k_{B}T}{\eta }L^{*}K\nabla c_{\infty }$$

where $k_{B}$ is the Boltzmann's constant, *T* is the absolute temperature, *η* is the fluid viscosity, $L^{*}$ and *K* are two parameters representing the solute profiles, and $\nabla c_{\infty }$ is the concentration gradient of the undisturbed solute concentration.

#### 2.2.2 Designs of Self‐Diffusiophoretic Swimmers

Based on this finding, Golestanian et al. proposed a theoretical model for these small particles.^[^ ^50^ ^]^ This was the first model to describe the motion of small particles in self‐generated solute gradients as, previously, only the small particles in externally generated solute gradients had been analyzed. An example of a simple machine utilizing this self‐diffusiophoretic propulsion mechanism is a spherical particle that has an asymmetrical small catalytic reaction site (Figure 4). Uncharged neutral solute molecules dissolved in a liquid interact with the surface of the catalytic site on the particle and are decomposed into several products. This increases the quantity of these products near the catalytic site and leads to an asymmetrical distribution of products around the particle. Owing to this gradient, the swimmers are pulled toward the region with a lower solute concentration.

**Figure 5:**
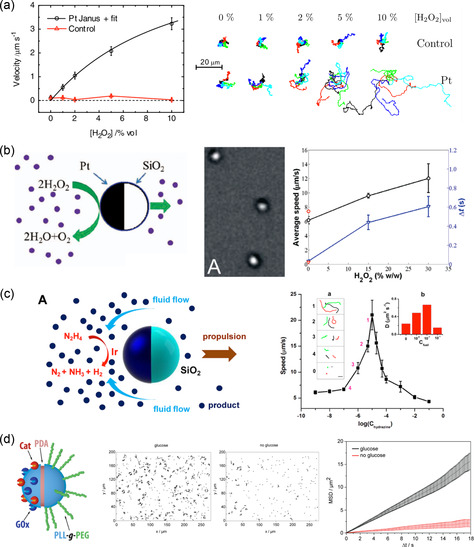
Spherical catalytic micro‐ and nanoswimmers propelled by the self‐diffusiophoresis mechanism. a) The velocity and trajectories of the microswimmer (polystyrene microbeads half coated with platinum) compared to those of the control (polystyrene microbeads) at various H_2_O_2_ concentrations. Adapted with permission.^[^ ^21^ ^]^ Copyright 2007, American Physical Society. b) A scheme of Pt‐coated SiO_2_ microswimmers moving directly to the noncatalytic site in H_2_O_2_ solutions, optical microscopy images of the Janus particle, and the average speed as a function of fuel concentrations. Left: Adapted with permission.^[^ ^111^ ^]^ Copyright 2014, American Institute of Physics. Right: Adapted with permission.^[^ ^23^ ^]^ Copyright 2010, American Chemical Society. c) A scheme of ${\text{Ir/SiO}}_{2}$ Janus micromotors powered by hydrazine (N_2_H_4_) and the average speed as a function of hydrazine concentration over the $10^{-9}-10$% range. Adapted with permission.^[^ ^24^ ^]^ Copyright 2014, American Chemical Society. d) A scheme of the assembled SiO_2_/PDA/PEG‐PLL/GOxCAT Janus particle and its motion trajectories and velocity in a fuel solution with/without glucose. Adapted with permission.^[^ ^51^ ^]^ Copyright 2015, American Chemical Society.

This theoretical model was experimentally verified by Howse et al.;^[^ ^21^ ^]^ they chose polystyrene spheres with a diameter of 1.62 μm as the microscale swimmer and coated one side of the spheres with platinum (Pt). The deposited Pt layer decomposes the H_2_O_2_ molecules (the fuel solution) into oxygen and water. This produces more molecules than those consumed as fuel near the Pt‐coated side of the microswimmer. **Figure** **5**a shows the experimental results of Pt‐coated Janus microbeads moving in the fuel solution. The propulsion velocity of the catalytic microswimmer is presented as a function of the H_2_O_2_ concentration and is compared to the velocity of the polystyrene microbeads, which serve as a control. The results show that a higher concentration of the fuel solution causes a higher velocity of the catalytic microswimmers, and a close‐to‐zero velocity was observed for the polystyrene microbeads without a catalyst.

The similar Pt‐coated Janus structure was also presented as a catalytic microswimmer driven by the self‐diffusiophoresis mechanism by Ke et al., however, they utilized silica (SiO_2_) as the body of the microswimmers instead of polystyrene.^[^ ^23^ ^]^ The experimental results revealed an increase in the average particle speed with increasing H_2_O_2_ concentrations, which is in accord with Howse's work (Figure 5b). They also analyzed the particle orientation with respect to the direction of motion and found that these two factors were highly correlated on short timescales. Gao et al. presented iridium‐based Janus microswimmers that can be powered by extremely low concentrations of chemicals.^[^ ^24^ ^]^ Silica particles with a diameter of 1.2 μm were coated with iridium metal and these Ir‐based Janus microswimmers could achieve a similar speed in a 0.001% N_2_H_4_ solution to Pt‐based microswimmers in a 10% H_2_O_2_ solution (Figure 5c). The Ir surface catalyzed the decomposition of hydrazine (N_2_H_4_) fuel into nitrogen gas (N_2_), hydrogen gas (H_2_), and ammonia (NH_3_). However, this also created a cationic species (${\text{NH}}_{4}^{+}$) as a result of the protonation of NH_3_ that drove the motion of the microswimmer by the diffusiophoretic mechanism due to nonelectrolyte and electrolyte products.

For biomedical applications, the biocompatibility and biotoxicity of the catalytic microswimmer, and its fuel solution must be considered; however, the commonly used fuel solutions H_2_O_2_ and hydrazine (N_2_H_4_) hinder the application of catalytic microswimmers in biomedicine owing to their cytotoxicity. Schattling et al., therefore, proposed the use of glucose as a fuel solution for catalytic microswimmers.^[^ ^51^ ^]^ Glucose is enzymatically degraded by glucose oxidase (GOx); however, this decomposition process again generates cytotoxic H_2_O_2_. The authors overcame this problem by designing Janus particles with GOx and catalase (Cat), which can enzymatically decompose H_2_O_2_ into water and oxygen. The Janus particles with GOx/Cat on one hemisphere and a diameter of 800 nm were used in the experiments. Figure 5d presents a scheme of the fabricated GOx/Cat Janus particles and their velocity and motion trajectories in HEPES buffer solution with 400 mm glucose, compared to those in pure HEPES buffer solution. NB: the issue of the fuel solution cytotoxicity is largely applicable to all the catalytic swimmers, and therefore, a similar approach has been adapted for other types of catalytic mechanisms.

**Figure 6:**
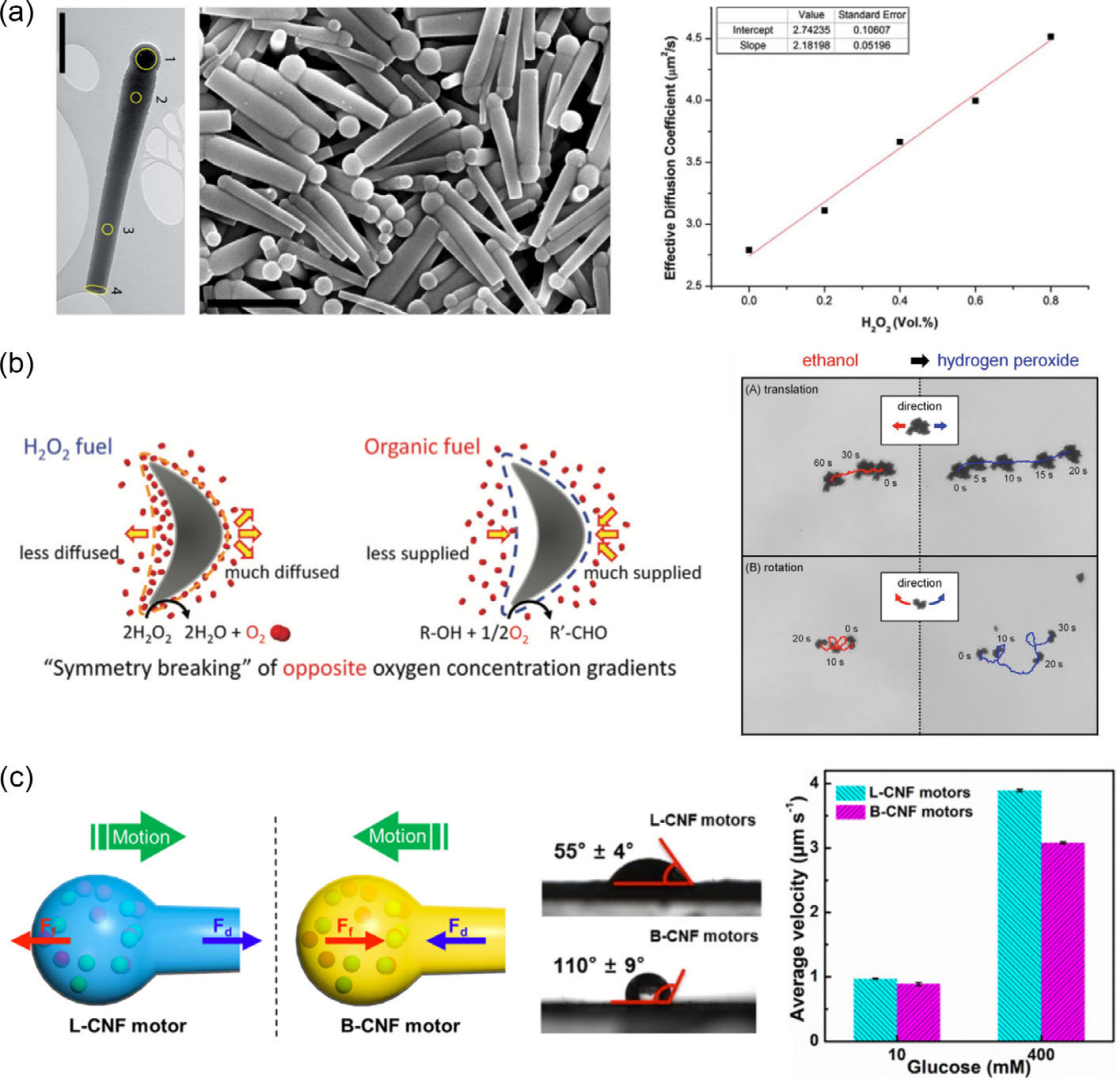
Catalytic micro‐ and nanoswimmers propelled by the self‐diffusiophoresis mechanism in a special shape. a) TEM and SEM images of the SiO_2_/Mn_*x*_O_*y*_ match‐like nanorod and its effective diffusion coefficient as a function of H_2_O_2_ fuel concentration. Adapted with permission.^[^ ^52^ ^]^ Copyright 2014, Royal Society of Chemistry. b) A scheme of the boomerang‐like microswimmer propelled in H_2_O_2_ and organic fuels, and its translational and rotational motion trajectories. Adapted under the terms of the CC‐BY Creative Commons Attribution 3.0 Unported license (https://creativecommons.org/licenses/by/3.0).^[^ ^26^ ^]^ Copyright 2015, Royal Society of Chemistry. c) A scheme of carbonaceous nanoflask (CNF) swimmers with different surface hydrophilicities (the hydrophilic CNFs are denoted as L‐CNFs and the hydrophobic CNFs as B‐CNFs), the water contact angle measurement for the two types of swimmers, and the comparison of the average velocities between swimmers. Adapted with permission.^[^ ^54^ ^]^ Copyright 2019, American Chemical Society.

A spherical Janus particle was proposed as the most popular design for catalytic microswimmers driven by a self‐diffusiophoresis mechanism, as it generates asymmetric solute gradients around the particle. However, other designs of catalytic micro‐ and nanoswimmers that are propelled by means of self‐diffusiophoresis have also been proposed recently. Morgan et al. proposed a silica‐manganese oxide (SiO_2_/Mn_*x*_O_*y*_) nanomachine in a matchstick‐like shape;^[^ ^52^ ^]^ the structure was fabricated with SiO_2_ and the head was coated with Mn_*x*_O_*y*_ (**Figure** **6**a). This match‐like nanobot with a length of 1.78 μm and a radius of 300 nm can move autonomously in an H_2_O_2_ solution owing to the phoretic force caused by the chemical gradient resulting from the decomposition of H_2_O_2_ occurring on the manganese oxide layer. The experimental results in Figure 6a show an increase in the effective diffusion coefficient of the nanorod with increasing H_2_O_2_ concentration. Yamamoto et al. proposed a boomerang‐like platinum (Pt) microswimmer that was propelled by the oxidation of specific organic compounds and compared its performance in H_2_O_2_ fuel.^[^ 26, 53 ^]^ By designing the morphology of the assembly of Pt particles, they could achieve directional motion using a single metal material. Figure 6b shows the translational and rotational motion trajectories of the proposed microswimmer in nontoxic fuels (e.g. 1% ethanol), and in 1% H_2_O_2_ fuel. Glucose‐powered microswimmer in a specially shaped nanoflask were also proposed, and their selective directional propulsion was proposed by utilizing their surface wettability.^[^ ^54^ ^]^ Gao et al. synthesized the hydrophilic carbonaceous nanoflask (L‐CNFs) by using a soft template‐based polymerization method. A further carbonizing treatment changed the surface property into a hydrophobic one (B‐CNFs). The catalytic components, GOx and Cat, were encapsulated in the cavity of the structure, which allows molecular diffusion through the narrower tubular neck of the nanoflask shape of the microswimmer. The opposite surface wettability of L‐CNFs and B‐CNFs lead to the opposing propulsion force, resulting in the opposing propulsion direction. The experimental results in Figure 6c reveal that the average velocities of L‐CNFs driven by the local glucose concentration gradient were relatively higher than those of B‐CNFs driven by the produced glucose acid gradient.

### 2.3 Bubble Recoil Swimmers

The bubble recoil mechanism that occurs in catalytic swimmers is predominantly observed in designs that include tubular and Janus microstructures. Therefore, in this section, we first discuss the bubble recoil mechanism of catalytic swimmers with a tubular structure and we explain the factors that can influence their dynamics, such as the material composition and/or the geometrical features. Next, we describe the motion and dynamic characteristics of Janus microswimmers and the factors that influence their locomotion patterns.

#### 2.3.1 Fundamentals of Bubble Recoil Locomotion Mechanism: Tubular Structures

**Figure 7:**
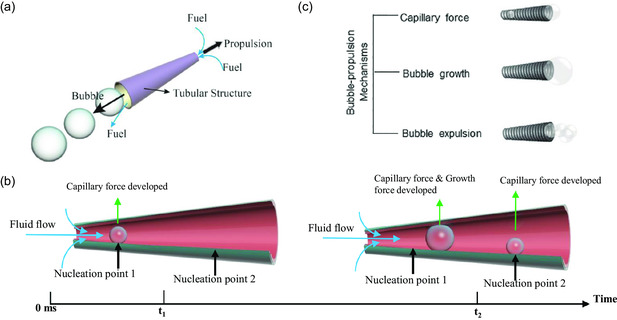
Bubble recoil mechanism of a tubular‐based swimmer. a) Schematic illustration of the Bubbles eject from the hollow microstructure that leads to its final movement. b) Schematic illustration of the bubble formations at nucleation points within a tubular‐based swimmer and the development of capillary force and growth force. c) The three stages of the bubble recoil mechanisms. Adapted with permission.^[^ ^59^ ^]^ Copyright 2021, The Authors, published by Wiley‐VCH.

Tubular micro‐ and nanostructures with either cylindrical or conical shapes can form and eject bubbles by chemical reaction with surrounding liquid to be propelled. Specifically, the chemical decomposition of fuel solutions occurs on the catalytic inner layer located inside the hollow structure and generates bubbles as a byproduct. When a bubble generates and grows to a certain size inside the hollow structure, it facilitates the emergence of capillary force. The capillary force pushes the bubbles forward along the hollow structure. During this movement, the bubbles produced can merge and grow until they are ejected from the end of the structure. As the bubbles are ejected from one opening of the tubular‐based swimmers, liquid is simultaneously drawn into another opening of the hollow structure (**Figure** **7**a).^[^ ^55^ ^]^ Accordingly, the net momentum of the fluid going in and out of the hollow structure leads to the generation of a jet force on the microstructure $F_{\text{jet}}$ (Equation (11)).^[^ ^56^ ^]^ Note that when a tubular‐based swimmer with a cylindrical shape undergoes a pressure difference, one of the two openings of the swimmer arbitrarily becomes an ejection port for the bubble release. However, for a conical‐shaped tubular swimmer, only the larger opening serves as the outlet for the bubble ejection.

(11) $$F_{\text{jet}}=\frac{1}{q}(|\frac{dm_{1}}{dt}|v_{f1}-|\frac{dm_{2}}{dt}|v_{f2})$$

where $v_{f1}$, $m_{1}$, and $v_{f2}$, $m_{2}$ represent the velocity and mass of fluid at the large and small openings, respectively. $|\frac{\left(dm_{1}\right)}{dt}|v_{f1}-|\frac{dm_{2}}{dt}|v_{f2}$ is the momentum difference between the small and the large opening, and *q* is the momentum transferred to the channel. After the bubble is ejected from the channel, the jet force vanishes. The bubble ejection process leads to the displacement of the tubular‐based swimmer in the opposite direction to that of the bubble ejection.^[^ 57, 58 ^]^

$F_{\text{jet}}$ relates to the velocity of the fluid and mainly depends on the size and growth rate of the bubbles. The capillary and growth forces that determine the jet force are influenced by the nucleation site of the bubbles in the channel. For instance, the closer the nucleation point is to the small opening, the quicker the bubbles make contact with the channel wall and the earlier the capillary and growth forces are developed in the channel (Figure 7b).^[^ ^56^ ^]^ At a low Reynolds number, the instantaneous resistance of $F_{\text{drag}}$ compensates for the driving $F_{\text{driving}}$ of the small swimmers, which is balanced with the linear drag force described in the Stokes drag equation. The dynamics of the micro‐ and nanoswimmers can be approximately described as^[^ 56, 58 ^]^

(12) $$F_{\text{jet}}=F_{\text{drag}}=\frac{2\pi \mu LV}{\ln(\frac{2L}{R})-0.72}$$

where *μ* is the fluid viscosity, *R* and *L* represent the radius and the length of the tube, and *V* is the velocity of the swimmer, respectively. From this equation, once $F_{\text{jet}}$ is known, the swimmer's velocity can be easily obtained.

The propulsion of tubular‐based swimmers consists of three stages (capillarity, bubble growth, and bubble expulsion), as depicted in Figure 7c.^[^ ^59^ ^]^ The capillarity stage is the condition where the bubbles move through the hollow structure while maintaining contact with the inner wall. In the growth stage, bubbles reach and adhere to the larger opening increasing their size until the bubbles detach from the tube opening and are ejected. The driving forces of tubular‐based swimmers in these three stages are the capillary force, growth force, and jet force, respectively. These driving forces are influenced by various factors, such as fuel concentration, surface tension, and medium viscosity,^[^ ^59^ ^]^ resulting in the different dynamics of the tubular‐based swimmers’ motion. At a high concentration of fuel, a rapid bubble generation and ejection can be accomplished.^[^ 60, 61 ^]^ However, when the surface tension of the medium is large, the moving speed of the bubble in the tube is slow, and therefore, the ejection rate of bubbles at the opening is also low.^[^ 56, 62 ^]^ To reduce the surface tension of the medium, additives, such as anionic surfactants (such as sodium dodecyl sulfate (SDS)), can be employed.^[^ ^63^ ^]^ In addition, as the medium viscosity increases, the residence time of the bubbles in the tube also increases, resulting in a reduced bubble ejection.^[^ ^64^ ^]^ Different concentrations of methylcellulose (MC) can improve the viscosity of the medium.^[^ 65, 66 ^]^

#### 2.3.2 Designs of Bubble Recoil Locomotion Mechanism: Tubular Microstructures

**Figure 8:**
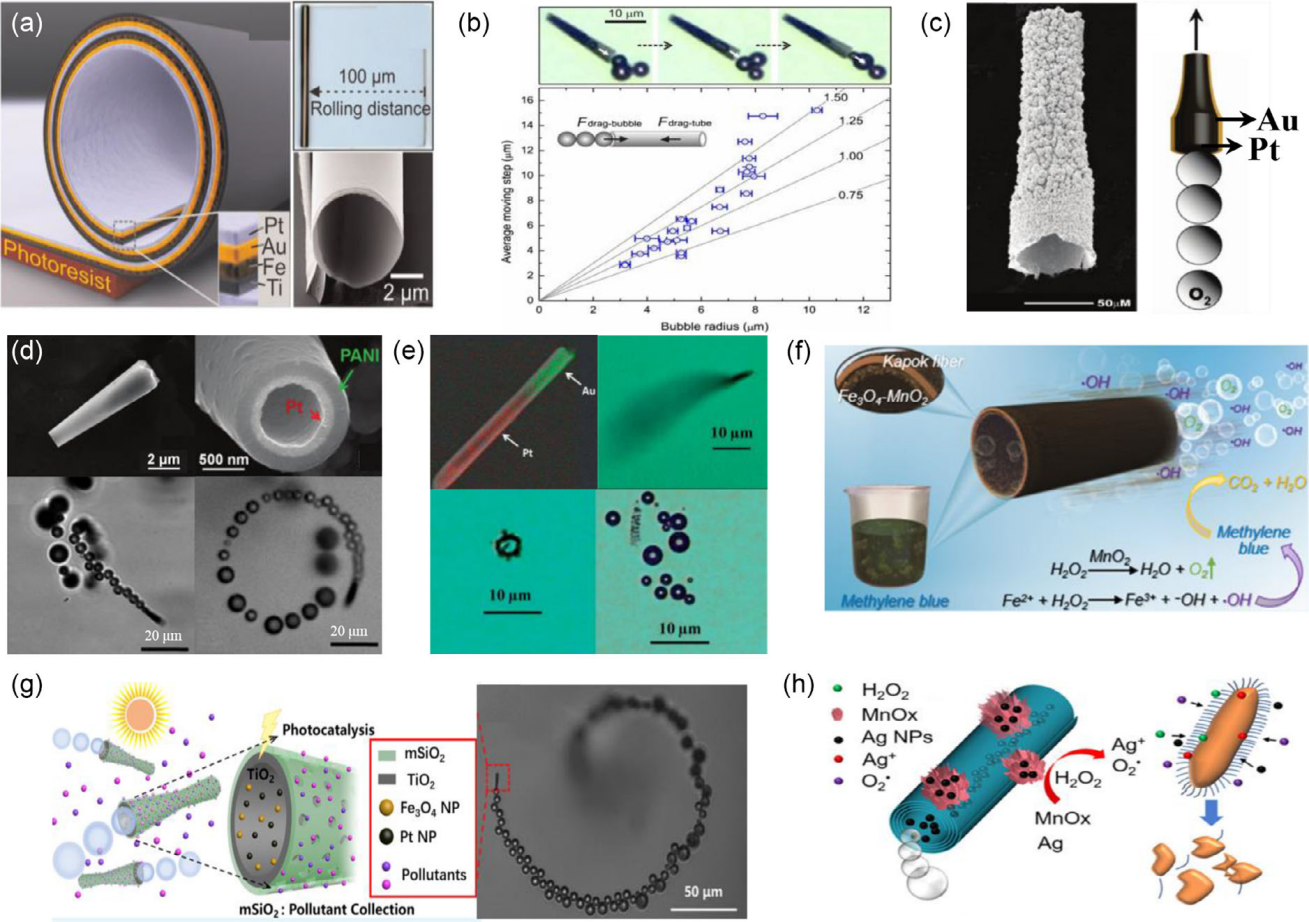
Various types of tubular microswimmers, propelled by a bubble recoil mechanism. a) A schematic illustration showing the rolling‐up approach. b) Microscopy images and a graph illustrating the average moving step as a function of the bubble radius when the bubble is released from the microtube. a,b) Adapted with permission.^[^ ^68^ ^]^ Copyright 2009, Wiley‐VCH. c) SEM images of Au/Pt conical microtubes together with a schematic illustration. Adapted with permission.^[^ ^63^ ^]^ Copyright 2010, American Chemical Society. d) SEM images and motions of the PANI/Pt microtube. Adapted with permission.^[^ ^69^ ^]^ Copyright 2011, American Chemical Society. e) SEM‐EDX elemental mapping and different motion modes of the Au/Pt nanotubes: straight, circular, and spiral. Adapted with permission.^[^ ^70^ ^]^ Copyright 2013, Royal Society of Chemistry. f) A schematic illustration of bubble‐driven MnO_2_/KF microtubes. Adapted with permission.^[^ ^71^ ^]^ Copyright 2021, American Chemical Society. g) A schematic illustration of a double‐layered tubular microswimmer and a video snapshot of it swimming in H_2_O_2_. Adapted with permission.^[^ ^72^ ^]^ Copyright 2018, American Chemical Society. h) A schematic illustration showing an Ag and manganese‐modified microtubule used for killing bacteria. Adapted with permission.^[^ ^73^ ^]^ Copyright 2022, Elsevier.

A variety of fabrication methods have been developed to optimize the structure of tubular‐based swimmers, as this plays a crucial role in their dynamics. Mei et al. and Solovev et al. fabricated the first example of a tubular‐based swimmer in 2005.^[^ 67, 68 ^]^ The swimmers were developed utilizing a roll‐up technology that exploits the internal stress among different deposited metallic layers. The lattice mismatch between the metallic layers facilitates the coiling of the layered structure when released from the surface. (**Figure** **8**a). In this study, the authors fabricated Ti/Fe/AuPt tubular‐based swimmers that could propel in H_2_O_2_ due to the continuous ejection of microsized bubbles formed on the catalytic inner Pt layer. Interestingly, it was also demonstrated experimentally in this work that the net displacement (or average moving step) of these tubular‐based swimmers increased by increasing the radius of the bubble being released (Figure 8b).

This pioneering work accelerated research in this type of tubular‐based swimmers and different geometries and smaller sizes were also analyzed. For example, Manesh et al. demonstrated that it is possible to fabricate conical microtubes via a sequential deposition of Pt and Au on a partially‐etched Ag wire that it is used as a sacrificial layer (Figure 8c).^[^ ^63^ ^]^ The degree of the taper in the conical microtubes could be tuned by adjusting the etching time of the Ag wire. In addition, Gao et al. fabricated polyaniline (PANI)/Pt conical microtubes following a sequential deposition of PANI and Pt into conically‐shaped micropores of ion‐track etched polycarbonate membranes (Figure 8d).^[^ ^69^ ^]^ Tubular‐based swimmers have been also fabricated with nanoscale dimensions. For example, Zhao et al. generated Au/Pt nanotubes by sequentially electrodepositing Au and Pt onto a porous alumina membrane with a pore size of 200 nm (Figure 8e).^[^ ^70^ ^]^ The visible bubbles released from the nanotubes were proof of the bubble recoil mechanism. However, porous membranes are not the only solution for templates to fabricate tubular‐based swimmers. For example, Chen et al. demonstrated that a catalytic tubular‐based swimmer could also be produced in a simple and easy manner by employing a biological template. In this study, the authors deposited manganese dioxide (MnO_2_) onto the inner and outer walls of kapok fiber (KF) matrices. MnO_2_ decomposes H_2_O_2_ to produce oxygen bubbles, thereby, promoting the movement of the swimmer (Figure 8f).^[^ ^71^ ^]^ Double‐layer tubular‐based swimmers have been also demonstrated with the incorporation of photocatalytic materials. For example, Liang et al. prepared mesoporous titanium dioxide double‐layer microtubules using a template‐assisted method where Pt and Fe_3_O_4_ nanoparticles were encapsulated in the structure. The Pt and Fe_3_O_4_ nanoparticles present in the tube catalyzed H_2_O_2_ to produce bubbles (Figure 8g).^[^ ^72^ ^]^ Although this article only discussed the photodegradation effect, the photocatalytic materials employed (i.e., the magnetic Fe_3_O_4_ nanoparticles and TiO_2_ inner layer) may affect the bubble formation under light illumination and further the dynamics of the swimmer. Moreover, Wang et al. proposed a hollow microtubule loaded with Ag and MnOx nanoparticles. Here, Ag and MnOx are used as catalysts to decompose H_2_O_2_, thereby producing a stronger synergistic driving force. In addition, the byproducts of reactive oxygen and Ag+ also help to rapidly kill bacteria (Figure 8h).^[^ ^73^ ^]^

#### 2.3.3 Fundamentals of Bubble Recoil Locomotion Mechanism: Janus Microstructures

Janus‐based swimmers are asymmetric structures that consist of a catalytic side and a noncatalytic side. Janus microstructures often exhibit a rapid locomotion that is triggered by the release of bubbles on the catalytic side in the presence of a fuel. It is generally accepted that Janus microspheres larger than 5 μm in diameter can move via a bubble recoil mechanism due to their small curvature, which facilitates a condition for bubble growth.^[^ 14, 74 ^]^ (NB: the spheres with <5 μm have a large curvature, which requires much more energy for bubble formation. Therefore, it is difficult for O_2_ to nucleate, grow, and separate. For this reason, the O_2_ byproducts usually exist as dissolved neutral molecules rather than bubbles. Consequently, this case follows the self‐diffusiophoresis mechanism as discussed in 2.2.)

**Figure 9:**
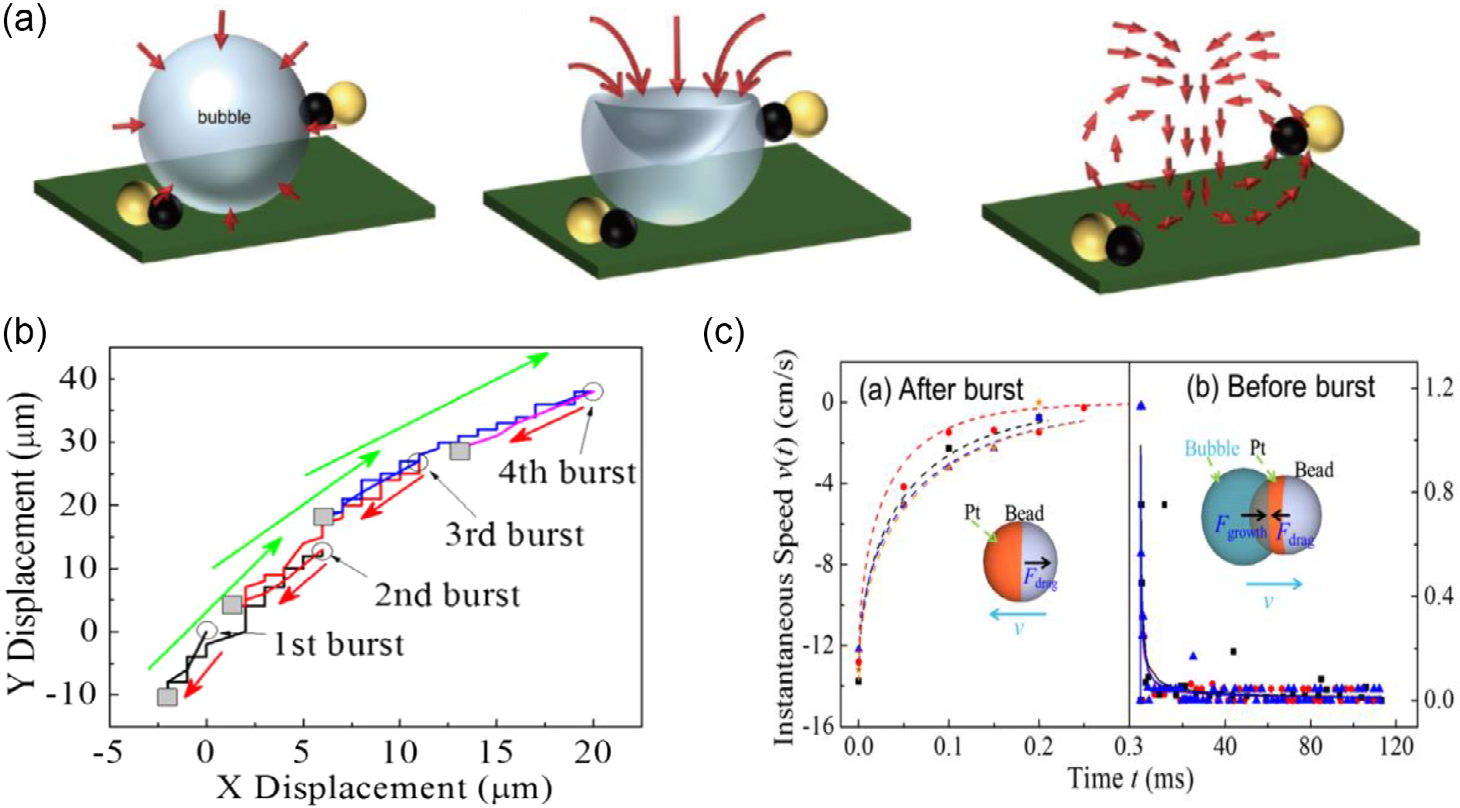
Bubble recoil mechanism of catalytically driven Janus microswimmers. a) A schematic diagram of the process from the collapse to the disappearance of the bubbles. Adapted with permission.^[^ ^75^ ^]^ Copyright 2014, American Chemical Society. b) Bubble generation and rupture result in a forward net displacement of the Janus microswimmer. c) Forces acting on a microswimmer in two bubble‐recoil stages (before and after bursting). b,c) Adapted with permission.^[^ ^74^ ^]^ Copyright 2012, American Physical Society.

During the process of bubble growth, the bubble can lift the swimmer. The center of the bubble can then move toward the center of the swimmer, thereby pushing it forward. However, when the bubble is released, a microjet develops on the releasing location, which leads to the generation of a vortex ring. The vortex ring allows an inward water flow that moves the Janus microspheres toward the center of the bubble, resulting in an opposite displacement (**Figure** **9**a).^[^ ^75^ ^]^ The overall displacement produced in the growth process is greater than that of the burst (or ejection) process, so that the Janus microswimmer has a net displacement in the forward direction (Figure 9b).^[^ ^74^ ^]^ The growth and collapse phenomenon of the bubbles appearing in the Janus microswimmers can be explained by the following formula^[^ 74, 76, 77 ^]^

(13) $$P_{b}-P_{\infty }=\frac{2\sigma }{R_{b}}+\frac{4\eta }{R_{b}}{\dot{R}}_{b}+\rho (R_{b}{\ddot{R}}_{b}+\frac{3}{2}{\dot{R}}_{b}^{2})$$

where $P_{b}$ and $P_{\infty }$ are the internal pressure of the bubble and the fluid pressure at a distance to the bubble, respectively. *σ*, *η*, and *ρ* represent the surface tension, viscosity, and density of the solution, respectively. $R_{b}$ is the radius of the bubble. As the bubble radius $R_{b}$ is very small, the bubble pressure is dominated by the viscous term, $P_{b}\approx 4\eta {\dot{R}}_{b}/R_{b}$. With the growth of the bubble, the radius increases gradually and the bubble pressure changes depending on the surface tension, $P_{b}\approx 2\sigma /R_{b}$. When the bubble grows to a certain size, the bubble pressure approximately equals the adjacent fluid pressure ($P_{b}\approx P_{\infty }$). In the process of bubble growth, the driving force of the Janus swimmer is the difference between the thrust of bubble growth and the resistance in the solution, $F=F_{\text{growth}}-F_{\text{drag}}$ ^[^ ^74^ ^]^

(14) $$F_{\text{growth}}=\pi \rho R_{b}^{2}(\frac{3}{2}C_{s}{\dot{R}}_{b}^{2}+R_{b}{\ddot{R}}_{b}),\;F_{\text{drag}}=6\pi \eta R_{m}v$$

where $C_{s}$ is an empirical constant, *v* and $R_{m}$ represent the speed and radius of the swimmer, respectively. $R_{b}$, *η*, and *ρ* are the radius of the bubble, and the viscosity and density of the fluid, respectively (Figure 9c).^[^ ^74^ ^]^

When the bubble grows to its maximal size before bursting, the bubble pressure equals approximately that of the adjacent fluid pressure ($P_{b}\approx P_{\infty }$). However, when the bubble bursts, it immediately reaches $P_{b}<P_{\infty }$ due to the sudden shrinkage of the bubble. Therefore, the swimmer is pulled back.^[^ 76, 77 ^]^ In this situation, the movement is governed by the drag force $F_{\text{drag}}$ as

(15) $$F_{\text{drag}}=6\pi \eta R_{m}v[1+\frac{R_{m}}{\sqrt{\pi vt}}+\frac{9}{16}\frac{R_{m}}{\lambda }(\frac{vt}{3\lambda^{2}})]$$

where *v* and *λ* represent the kinematic viscosity and the vertical distance from the center of the sphere to the wall, respectively.

In conclusion, the net displacement of Janus microswimmers is triggered by the competition between the forward propulsion mechanism caused by the bubble growth and the backward dragging force created by the bubble burst. These complex dynamics contributes to the bubble recoil mechanism of Janus microswimmers.

#### 2.3.4 Designs of Bubble Recoil Locomotion Mechanism: Janus Microstructures

**Figure 10:**
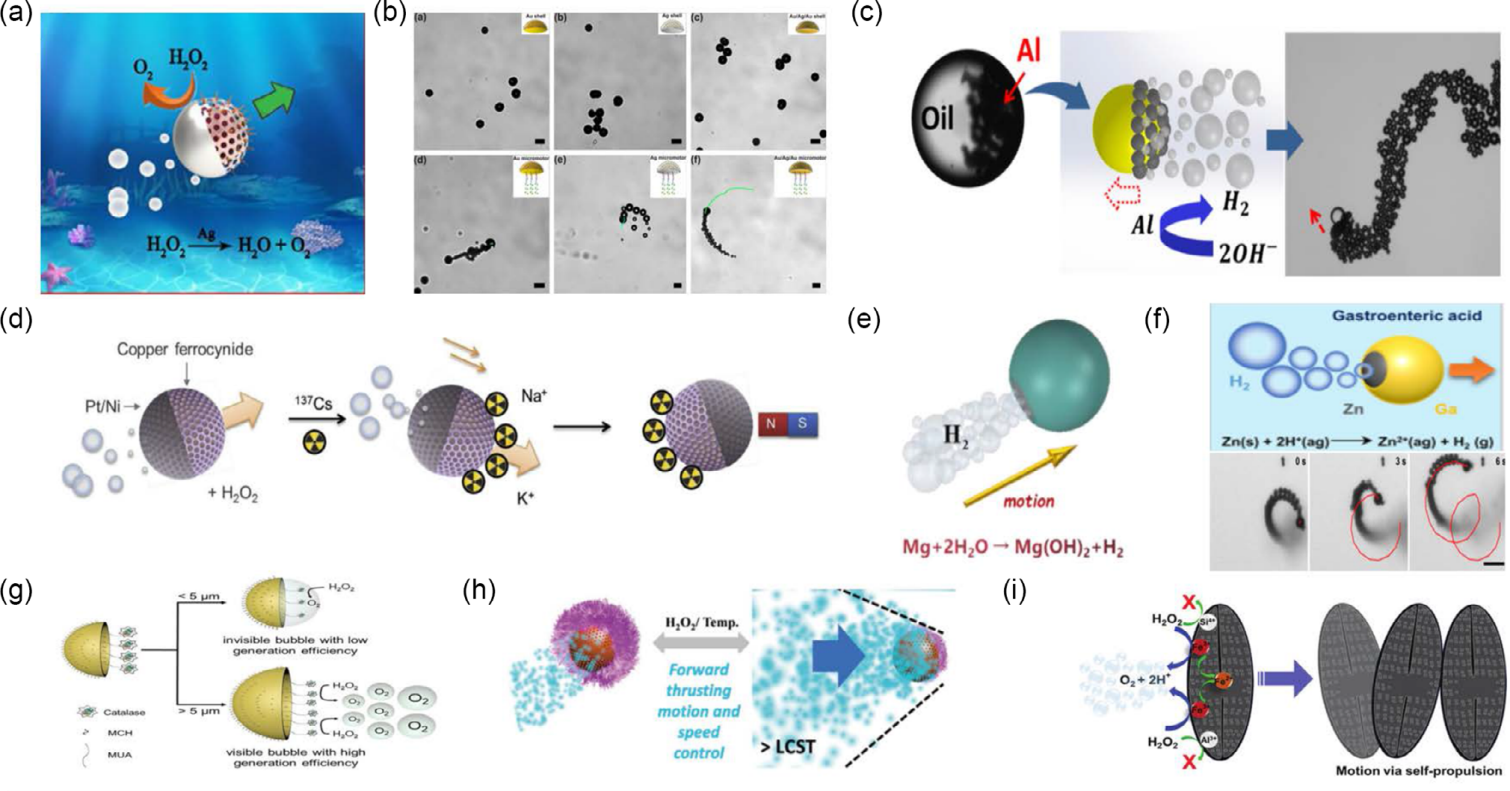
Various Janus microswimmers propelled by the bubble recoil mechanism. a) Schematic illustration of a Janus microswimmer that shows a Ag‐based catalytic motion. Adapted with permission.^[^ ^80^ ^]^ Copyright 2021, Elsevier. b) Microscopy images of Au/Ag/Ni/Au modified jellyfish‐like microswimmers with a polymetallic shell. Adapted with permission.^[^ ^81^ ^]^ Copyright 2019, American Chemical Society. c) Schematic illustration of a Janus microswimmer that shows an Al‐based catalytic motion in alkaline medium. Adapted with permission.^[^ ^82^ ^]^ Copyright 2018, Elsevier. d) Schematic illustration of a Janus microswimmer that shows a Pt/Ni‐based catalytic motion and its application for removing radioactive cesium. Adapted with permission.^[^ ^83^ ^]^ Copyright 2019, Elsevier. e) Schematic illustration of a Janus microswimmer that shows a motion based on Mg‐H_2_O_2_ reaction. Adapted with permission.^[^ ^86^ ^]^ Copyright 2021, Elsevier. f) Kinematic mechanism diagram and actual optical images of a Ga/Zn Janus microswimmer within gastric acid environment. Adapted with permission.^[^ ^87^ ^]^ Copyright 2021, Wiley‐VCH. g) Motion mechanism of microswimmers powered by catalase. Adapted with permission.^[^ ^14^ ^]^ Copyright 2019, Wiley‐VCH. h) Schematic illustration of a catalytic‐driven microswimmer where the speed is controlled by changing temperature. Adapted with permission.^[^ ^92^ ^]^ Copyright 2021, Wiley‐VCH. i) Schematic illustration of a diatom microswimmer that shows a Fe_2_O_3_‐based catalytic motion. Adapted with permission.^[^ ^93^ ^]^ Copyright 2018, Royal Society of Chemistry.

Chi et al. concluded that the bubble recoil process of Janus swimmers can be developed with a variety of fuels, including acid and/or alkali solutions, water, and H_2_O_2_.^[^ 78, 79 ^]^ A Janus microswimmer composed of mesoporous silica was proposed for environmental remediation by Yang et al.^[^ ^80^ ^]^ The coated Ag layer catalyzes the fuel solution (H_2_O_2_) and produces oxygen bubbles (**Figure** **10**a). This reaction enables the net movement of the swimmer. In another example, a jellyfish‐like Janus microswimmer realizes linear, circular, and helical motion driven by oxygen bubbles in H_2_O_2_ solution at different concentrations. This microstructure demonstrates an efficient bubble propulsion mechanism even at low concentrations of H_2_O_2_. The jellyfish‐like microswimmer was obtained by attaching DNA onto the concave surface of a polymetallic Au/Ag/Ni/Au shell and then modifying the attached DNA with catalase. Indeed, by decreasing the opening size of the shell, the bubble generation is reduced, resulting in a reduction in the speed of movement. When the DNA components on the jellyfish‐like Janus microswimmer hybridize to the target DNA in the environment, they are released from the swimmer together with the catalase modified on them, resulting in a reduction of the motion speed. Therefore, this approach enables sensitive detection of DNA solely by monitoring the speed of the jellyfish‐like Janus microswimmers (Figure 10b).^[^ ^81^ ^]^ In addition to the common solid particles, Li et al. introduced a Janus droplet microswimmer fabricated by dispersing an oil droplet containing Al particles into an aqueous solution. The Al particles gathered at the bottom of the oil droplet due to gravity, hence forming an Al/Oil Janus structure. In an alkaline medium, Al reacts with the alkaline solution to produce hydrogen bubbles and promote the movement of the droplet. The result shows that the propulsive force linearly and positively correlates with the number of bubbles separated per unit time (Figure 10c).^[^ ^82^ ^]^

Magnetic fields enable precise control of the swimmers and, therefore, allow for targeted specific treatments. Hwang et al. prepared a Janus microswimmer by depositing Ni and Pt on the surface of copper ferrocyanide functionalized on a mesoporous silica microsphere. The continuous release of bubbles that catalyzed on the Pt surface enabled the movement of the Janus structure. Moreover, they also demonstrated that the Janus microswimmer could adsorb and remove radioactive cesium from an aqueous solution. Indeed, under a magnetic field, the microswimmer could move rapidly in a biased direction, achieving a successful and efficient removal rate of radioactive cesium. These results could be of great interest for nuclear wastewater treatment (Figure 10d).^[^ 83, 84 ^]^ Most catalytic microswimmers require highly concentrated fuel solutions to have an efficient and stable propulsive force. However, this condition seriously limits the biomedical application of catalytic‐based microswimmers when the fuel solution is toxic.^[^ ^85^ ^]^ To tackle this problem, Feng et al. designed a microswimmer based on magnesium, which is a catalytic material that can perform in vivo navigation through the bubble recoil mechanism. In this work, the bubbles were generated by a spontaneous chemical reaction between magnesium and water (Figure 10e).^[^ ^86^ ^]^ Another Janus gallium/zinc (Ga/Zn) microswimmer that has shown a good performance for treating bacterial infections was presented by Lin et al. In this case, the Janus microstructure was propelled by hydrogen generated by a zinc acid reaction in a gastric acid environment (Figure 10f).^[^ ^87^ ^]^ In addition, a suction cup‐shaped magnesium microswimmer that continuously generates hydrogen under the action of gastric acid has been reported.^[^ ^88^ ^]^ Additionally, Xu et al.^[^ ^89^ ^]^ reported the observation of the bubble propulsion mechanism through water radiolysis in Cu/SiO_2_ Janus particles under X‐ray illumination. When these particles were exposed to X‐rays, microbubbles of H_2_ were generated specifically on the Cu side, owing to the higher transfer of radiation dose from Cu to water compared to SiO_2_ to water. Traditionally, swimmers propelled by photocatalytic reactions have primarily relied on UV–vis‐NIR light sources.^[^ 90, 91 ^]^ However, these light sources have limited penetration depths, which impose significant restrictions for in‐vivo applications. In contrast, the utilization of X‐rays, which possess superior penetration depths, represents a novel approach for light‐induced bubble propulsion, particularly in clinical applications.

Moreover, spherical Janus swimmers can be also chemically modified by functionalizing the outermost catalytic layer. For example, Chen et al. proposed a multimetallic Au/Ag/Au microshell prepared using the template directional deposition method.^[^ ^14^ ^]^ This multimetallic swimmer was modified with catalase on the concave surface of the microshell, a feature that enabled the fabrication of Janus enzyme‐powered microswimmers. In this study, the authors revealed the influence of the fuel, the concentration, and the size of swimmer on the dynamics of the designed Janus enzyme‐powered microswimmer. It was demonstrated that these Janus enzyme‐powered microswimmers could achieve a net motion even at a very low concentration of H_2_O_2_ if the well‐tailored geometry is optimized (Figure 10g).^[^ ^14^ ^]^ It is also worth noting that a reversible microswimmer braking system was proposed. The intelligent Janus microswimmer was obtained by grafting a heat‐sensitive polymer polyphosphazene (PPz) onto mesoporous organosilica microparticles internally loaded with Mn. When the temperature of the solution drops below the lower critical transition temperature of the polymer, the collapse/expansion of the polymer prevents H_2_O_2_ from entering the pores of the Janus microswimmer. This reduces the generation of bubbles, realizing the motion switching by decreasing the velocity (Figure 10h).^[^ ^92^ ^]^ In addition to the catalysts commonly used to achieve a bubble‐recoil propulsion (such as Pt, Ag, and Mn), some research groups have considered using other types of catalysts to design safer and more environmentally friendly microswimmers. For example, in Panda's work on bioinspired microswimmers based on activated diatoms containing Fe_2_O_3_, significant bubble propulsion was achieved at a very low H_2_O_2_ concentration (0.8%), as Fe_2_O_3_ acts as the catalyst for the H_2_O_2_ decomposition.^[^ ^93^ ^]^

### 2.4 Multilocomotive Swimmers

#### 2.4.1 Fundamentals of Multilocomotion Mechanism

So far, we have discussed three propulsion mechanisms of catalytically propelled swimmers, providing examples of the motion dynamics of new structural and material designs for these swimmers. The advantages of using chemical energy to generate mechanical energy in small‐scale agents, as opposed to relying on external energy sources, are cost‐effectiveness and the ability to operate in unlimited working spaces, assuming a suitable fuel solution is provided. Additionally, the collective behavior observed in catalytic swimmers can serve as a model for understanding the fundamentals of collective behavior displayed by certain living entities, further emphasizing the merits of this field.^[^ 1, 94, 95 ^]^ However, a grand challenge in chemically driven micro‐ and nanoswimmers is achieving autonomous navigation in unpredictable environments, especially within the human body. Some studies have demonstrated programmed rotary motion by appropriately designing catalytic swimmers.^[^ ^96^ ^]^ Previous research has also shown the feasibility of single chemotactic events, enabling swimmers to move toward higher fuel concentrations of the surrounding environment.^[^ 52, 94, 97 ^]^ To achieve full autonomy, the next step is enabling directional changes in movement by adjusting the propulsion mechanism in response to the surrounding environment, similar to natural microorganisms, which exhibit dual chemotaxis (positive and negative chemotaxis) in response to chemical cues.

#### 2.4.2 Designs of Multilocomotive Swimmers

**Figure 11:**
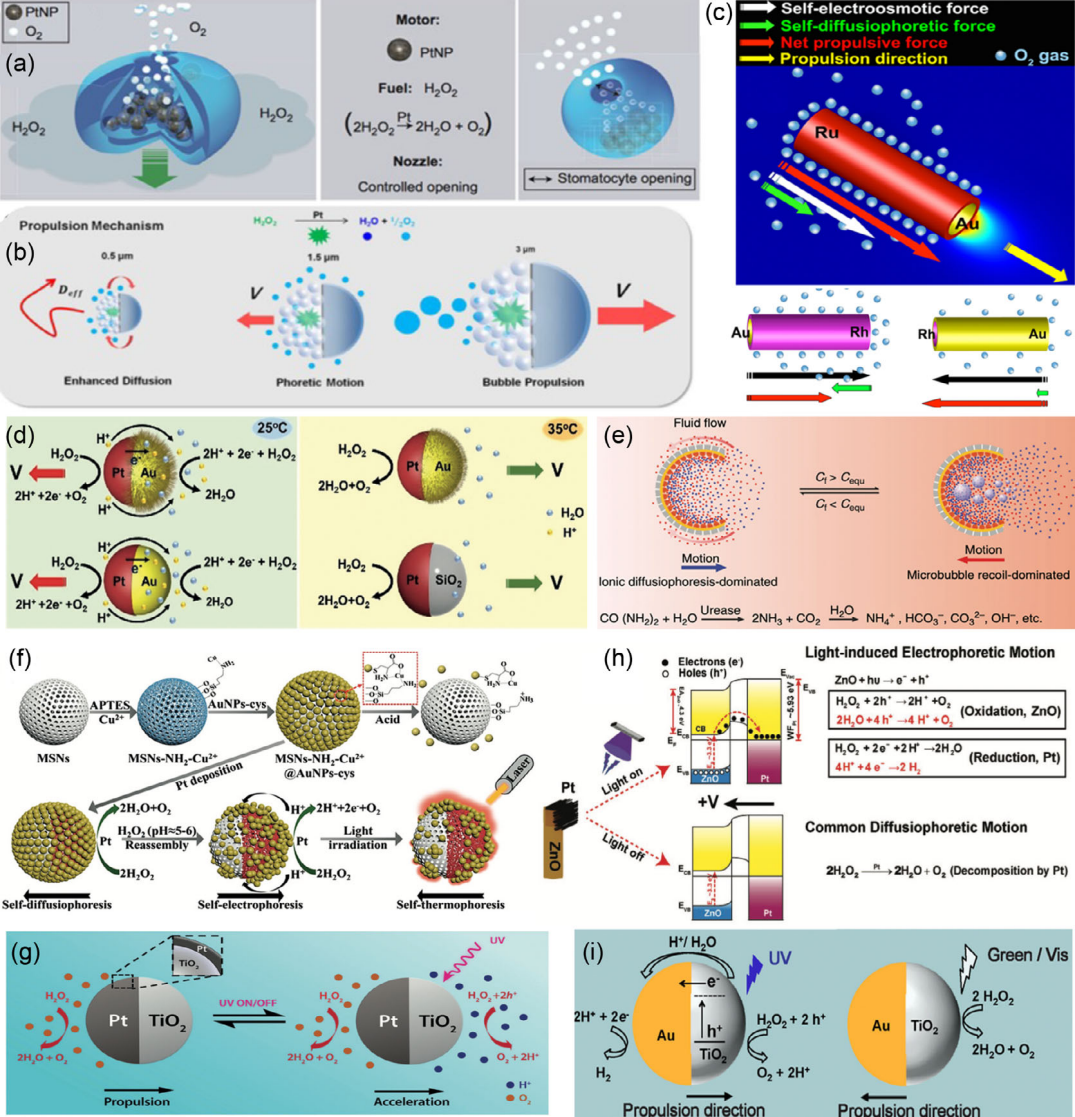
Multimechanisms in the micro‐ and nanoswimmers with complex architectures. a) Polymer stomatocytes entrapping Pt nanoparticles that show a dual mechanism. Adapted with permission.^[^ ^98^ ^]^ Copyright 2012, Springer Nature. b) Reversed Janus structures that show different mechanisms with the swimmer size. Adapted with permission.^[^ ^99^ ^]^ Copyright 2016, American Chemical Society. c) Core/shell bimetallic nanoswimmers that show different mechanism with material combinations. Adapted with permission.^[^ ^100^ ^]^ Copyright 2016, American Chemical Society. d) PNIPAM@Au‐Pt microspheres that show a change in the mechanism by the surrounding temperature. Adapted with permission.^[^ ^101^ ^]^ Copyright 2018, Wiley‐VCH. e) Pot‐like microswimmers that show a change in the mechanism with the urea concentration. Adapted with permission.^[^ ^102^ ^]^ Copyright 2021, American Physical Society. f) Janus mesoporous silica‐Pt@Au (JMPA) nanospheres that show a change in the propulsion mechanism with the local pH change. Adapted with permission.^[^ ^103^ ^]^ Copyright 2020, Wiley‐VCH. g) TiO_2_‐Pt microspheres and h) Pt‐ZnO nanorods that show a change in the mechanism with/without UV. Adapted with permission.^[^ ^104^ ^]^ Copyright 2018, Wiley‐VCH. Adapted with permission.^[^ ^105^ ^]^ Copyright 2019, Wiley‐VCH. i) Au‐TiO_2_ microspheres that show a change in the mechanism with the wavelength. Adapted under the terms of the CC‐BY Creative Commons Attribution 4.0 International license (https://creativecommons.org/licenses/by/4.0).^[^ ^106^ ^]^ Copyright 2020, The Authors, published by Springer Nature.

Recently, new advances made in the field of micro‐ and nanotechnology have enabled the design and fabrication of catalytic micro‐ and nanoswimmers with more complex architectures to achieve the tunability of propulsion mechanism. For example, Wilson et al. suggested that the locomotion mechanism of polymer stomatocytes containing Pt nanoparticles in their inner cavity (**Figure** **11**a) is due to both the rapid ejection of O_2_ through the stomatocyte opening and the chemical gradient generated around it.^[^ ^98^ ^]^ Sanchez et al. demonstrated that by changing the size of a reversed Janus mesoporous silica particle with Pt nanoparticles trapped inside the porous network, the mechanism can be modified from bubble recoil propulsion to phoretic and/or enhanced diffusive motion (Figure 11b).^[^ ^99^ ^]^ Enhanced‐diffusion, diffusiophoretic, and bubble propulsion mechanisms occur for Janus structures with sizes of 0.5, 1.5, and 3 μm, respectively, suggesting that one sole mechanism cannot explain the propulsion mechanism of certain systems. In addition, Jang et al. demonstrated unexpected propulsion dynamics of bimetallic nanoswimmers with a catalytic core/shell nanowire structure (Figure 11c).^[^ ^100^ ^]^ In contrast to the behavior of conventional bimetallic nanoswimmers, such as Au/Pt nanowires and Au/Pt spheres, the synthesized core/shell structure shows an increase in speed with an increase in its length. This can be explained by the concurrence of self‐electrophoresis and self‐diffusiophoresis owing to the nature of the catalyst, i.e., the Ru shell. Specifically, the bimetallic configuration causes a self‐electrophoretic force, while the Ru shell itself is capable of direct fuel decomposition, and therefore, develops a self‐diffusiophoretic force in the same direction as that of the self‐electrophoretic force. The increase in the length of the structure allows for the enlargement of the surface area of the Ru shell as well as the self‐diffusiophoresis, a feature that increases the speed of the swimmer. Other material combinations of core and shell layers, such as Au core/Rh shell and Rh core/Au shell, are used to prove the proposed propulsion mechanism, which shows that two forces can be either reinforced or deactivated, ultimately affecting the final speed of the swimmer.

Interestingly, Ji et al. reported a change in the propulsion mechanism of Janus Au/Pt bimetallic microswimmers functionalized with poly(*N*‐isopropylacrylamide) (PNIPAM) based on the surrounding temperature.^[^ ^101^ ^]^ The PNIPAM polymer brushes exhibit a phase transition between a hydrated state and a dehydrated state at the lower critical solution temperature (LCST). At the temperature below the LCST, PNIPAM brushes became hydrophilic and swollen, enabling the nanoswimmer to be propelled by the self‐electrophoretic mechanism, like Pt/Au Janus swimmers without functionalization. However, when the temperature exceeded the LCST, PNIPAM brushes turned hydrophobic and collapsed, minimizing the exposure area of the Au surface. This switches the governing driving mechanism of the brushes to self‐diffusiophoresis, like Pt/SiO_2_ swimmers. Feng et al. demonstrated a pot‐like microswimmer containing urease inside its confined cavity, exhibiting distinct motion mechanisms at different urea concentrations.^[^ ^102^ ^]^ At lower urea concentrations, urea is decomposed into CO_2_ and NH_3_, and further converted into ionic species (NH_4_^+^, ${\text{HCO}}_{3}^{-}$, CO_3_^2−^, OH^−^) in an aqueous solution, resulting in ionic diffusiophoresis. In contrast, at higher urea concentrations, the NH_3_ microbubbles are promoted, leading to motion predominantly driven by bubble recoil. Furthermore, Xing et al. presented pH‐responsive Janus mesoporous silica‐Pt@Au (JMPA) nanoswimmers, demonstrating their capability to transition between self‐diffusiophoretic and self‐electrophoretic propulsion mechanisms.^[^ ^103^ ^]^ Initially, the JMPA nanoswimmers exhibit self‐diffusiophoretic propulsion in a 3.0% H_2_O_2_ solution. However, the vulnerability of AuNPs to acid detachment causes the higher local pH resulting from H_2_O_2_ decomposition on the Pt side, which triggers the reassembly of AuNPs. This reassembly leads to aggregation on the Pt side and enables self‐electrophoresis.

Such mixed mechanisms or changes in the propulsion mechanism are often found in certain swimmers through induced photocatalytic reactions upon light illumination. For instance, Chen et al. introduced a TiO_2_/Pt microsphere with dual propulsion mechanisms.^[^ ^104^ ^]^ Under normal conditions without UV light, Pt catalyzes the nonelectrochemical decomposition of hydrogen peroxide, resulting in the production of only neutral molecules of oxygen and leading to self‐diffusiophoresis. However, when exposed to UV light, TiO_2_ generates a number of photoinduced electron–hole pairs, enabling the electrochemical decomposition of hydrogen peroxide. The resulting local proton gradient develops self‐electrophoresis and adds to the force generated by the nonelectrochemical decomposition on Pt, resulting in an accelerated speed. Instead of TiO_2_, Ying et al. used the properties of photogenerated carriers generated by ZnO to develop a Pt‐ZnO rod swimmer capable of exhibiting distinct motion mechanisms under different conditions.^[^ ^105^ ^]^ In the presence of UV light, electrophoretic motion occurred, driven by the electric field generated by a proton gradient due to the electrochemical decomposition of H_2_O_2_ (oxidation on ZnO and reduction on Pt). In contrast, in the absence of UV light, diffusiophoretic motion appeared, driven by the nonelectrochemical decomposition of H_2_O_2_, which only took place on the Pt side. In a similar vein, Vutukuri et al. used the characteristics of photogenerated carriers to create an Au/TiO_2_ swimmer with the ability to move in response to different lighting conditions.^[^ ^106^ ^]^ Under the UV irradiation, photogenerated carriers of TiO_2_ facilitate the electrochemical decomposition of H_2_O_2_, thereby resulting in a self‐electrophoretic motion. According to the author, while under green or high‐intensity visible light illumination the weak photocatalytic nature of TiO_2_ induces a self‐diffusiophoretic motion. However, the information on how the induced photons decompose H_2_O_2_ is not clearly detailed in the article.

## 3 Conclusions and Perspectives

Despite recent technological developments in micro‐ and nanoswimmers, there still remain challenges that need to be addressed for their successful application in biology and medicine. These challenges include the need for increased propulsive force, real‐time monitoring technology, precise motion control methods, biodegradability, and noncytotoxicity. Researchers have explored various avenues, including the use of magnetic, electric, optic, chemical, acoustic propulsion, or combinations of these to address these challenges. The use of external sources for propulsion requires systems that will be able to transfer energy in large working spaces (e.g., the human body), which is a challenging endeavor. Chemically driven swimmers, in contrast, show autonomous motion without restrictions of working space, as long as enough fuel is supplied to create a chemical reaction for propulsion. Some studies have demonstrated the use of chemically driven swimmers in gastrointestinal diseases and wound healing.^[^ 107, 108, 109, 110 ^]^ However, despite these advancements, there are still bottlenecks that need to be overcome for their use in clinical applications. One major challenge is the cytotoxicity of chemical fuels and the use of nonbiodegradable materials, which limit the safety and long‐term applications of these devices inside living organisms. Some studies have shown promising results using safer fuels such as urea and H_2_O. Another significant issue is the lack of controllability resulting from the complex reaction kinetics of chemically driven swimmers. To address this, we emphasize the importance of developing multipropulsion mechanisms as microorganisms in nature demonstrate the capability of propulsive directional change through dual mechanisms known as positive and negative chemotaxis. With this perspective, we reviewed the fundamentals of three propulsion mechanisms (self‐electrophoresis, self‐diffusiophoresis, and bubble recoil) of catalytic micro/nanoswimmers, leading readers toward the concept of multilocomotive mechanisms as a solution to achieve fully autonomous navigation. Furthermore, we highlighted the importance of considering features such as geometry, material composition, and fuel solution, as they can influence the propulsion mechanism of these swimmers, as summarized in **Table** **1**. Understanding the dynamics of motion in these tiny structures is essential, as it enables precise manipulation in small and complex environments that are challenging for humans to access.

**Table 1:** A list of catalytic micro‐ and nanoswimmers, categorized by their propulsion mechanism

Propulsion mechanism	Shape	Material	Length [μm]	Fuel solution	Speed [μm s^−1^]	Speed [body length s^−1^]	Key fabrication method	References
Self‐electrophoresis	Solid rod	Au/Pt	2	5% H_2_O_2_	20	10	Template‐assisted electro‐deposition	[39]
		Rh/Au			23.8	11.9		
		Pd/Au			15.3	7.6		
		Pt/Ru			30.2	15.1		
		Au/Ru			24.0	12.0		
		Rh/Pt			17.0	8.5		
		Rh/Pd			16.2	8.1		
		Pt/Pd			13.6	6.8		
		Ni/Au			4.7	2.3		
		Au/Au			6.2	3.1		
		Au/Co			7.1	3.5		
		Au_75_Au_25_/Pt			87.2	43.6		[40]
		Pt‐CNT/Au			42.8	21.4		[41]
		Cu/Pt	3.6	0.2 mM Br_2_	7	1.9		[44]
				0.2 mM I_2_	12	3.3		
		Carbon fiber, coated with GOx and BOx	8 × 10^3^	10 mM glucose	100	1.2	Hydrophilic treatment	[46]
	Tubular rod	Au/Ru	3	5% H_2_O_2_	32	10.8	Template‐assisted electro‐deposition	[37]
	Solid sphere	Au/Pt	2	4% H_2_O_2_	15	7.5	Sputtering	[47]
			0.03	1.5% H_2_O_2_	660	2.2 × 10^4^	Glance angle deposition	[22]
Self‐diffusiophoresis	Solid sphere	Pt/polystyrene Janus	1.62	10% H_2_O_2_	3	1.85	Evaporation	[21]
		Pt/SiO_2_ Janus	1	27.3% H_2_O_2_	12	12	e‐beam evaporation	[23]
		Ir/SiO_2_ Janus	1.2	0.001% N_2_H_4_	20	16.67	Sputtering	[24]
		GOx&Cat /PLL‐PEG @PDA coated on SiO_2_	0.8	400 mm glucose solution containing 20 wt% glycerol	0.37b)	NAa)	Pickering emulsion, enzyme immobilization	[51]
	Match‐stick	SiO_2_/Mn_*x*_O_*y*_	1.7	0.8% H_2_O_2_	4.52b)	NAa)	Evaporation	[52]
	Boomerang	Pt particles	NAa)	1% ethanol	0.88b)	NAa)	Aggregation	[26]
				1% H_2_O_2_	0.93b)			
	Nanoflask	Carbonaceous nanoflask encapsulating GOx&Cat	0.844	10 mM glucose solution	3.89	4.6	Template‐based polymerization	[54]
Bubble recoil	Thin plate	PDMS/porous glass, coated with Pt	9 × 10^3^	At the H_2_O_2_/air interface (3% H_2_O_2_)	2 × 10^4^	2.2	Sputtering, manual assembling	[30]
		Ti/Fe/Cr/Pt	10	9% H_2_O_2_	180	18		[112]
	Tubular	rGO/Ni/PtNPs	10	1% H_2_O_2_, 1% NaCh	140	14	Template‐assisted electrodeposition	[113]
		PPy‐COOH/PPy/Ni/Pt	10.5	4% H_2_O_2_	491	46.7		[114]
		PEDOT/Ni/Pt	15	5% H_2_O_2_	696	46.4		[115]
		WS_2_/Pt	15	2% H_2_O_2_	130	8.7		[116]
		SW‐Fe_2_O_3_/MnO_2_	12	5% H_2_O_2_	910	75.8		[117]
		Ti/Fe/Cr/Pt	10	3% H_2_O_2_	74	7.4		[118]
		PEDOT/Pt	7.2	3% H_2_O_2_	360	50		[119]
		PANI/Zn	10	acid environment	1050	105		[15]
		Fe_3_O_4_‐Fe‐ZIF‐8‐Pt	10	5% H_2_O_2_	860	86	Template‐based method	[120]
		Ti/Cr/Pt	50	2% H_2_O_2_	65	1.3	Sputtering, e‐beam evaporation	[62]
		PLA/HSA/MNP(PLA/HSA)5PLA/PLG/Avi	24	2% H_2_O_2_	60	2.5	Layer‐by‐layer assembly	[121]
		g–C_3_N_4_	67	10% H_2_O_2_	72	1.07	Hydrothermal reactions	[122]
	Solid sphere	S‐CuFC/Ni/Pt	2	20% H_2_O_2_	101.8	50.9	Chemical modification, sputtering	[83]
		Fe_2_O_3_@Ag‐mSiO_2_‐NH_2_	0.56	10% H_2_O_2_	203.1	253.8	Solvothermal treatment	[80]
		ZIF‐8@ ZnONPs	241	30% H_2_O_2_	1109	4.6	Chemical reaction, sputtering	[123]
		polystyrene@ ZIFs‐Zn‐Fe	80	6% H_2_O_2_	623	7.8		[124]
		PS/NiFe	10	6.3% H_2_O_2_	3.3 × 10^3^	320	Electrodeposition	[125]
		ZnO/Pt	1.5	5% H_2_O_2_	350	233.3		[126]
		Ga/Zn	8.8	Gastroenteric fluid	383	43.5	Microcontact printing	[87]
		Poly(ETPTA) /Fe_3_O_4_/MnO_2_	200	20% H_2_O_2_	424	2.12	Template method, UV exposure	[127]
		Pt‐SiO_2_	50	3% H_2_O_2_	500	10	E‐beam evaporation	[128]
		UCNP/Pt	5	5% H_2_O_2_	110	22	Layer‐by‐layer assembly, chemical functionalization, sputtering	[129]
		Ir‐TiO_2_‐Ni‐Ti‐SiO_2_	1.2	0.001% hydrazine	21	17.5	Sputtering, deposition	[24]
		Am‐TiO_2_/Au	15	15% H_2_O_2_	135	9	Microemulsion, sputtering	[130]
		TiO_2_‐Fe	15	5% H_2_O_2_	260	17.3		[131]
Multimechanism	Stomatocyte	PEG/PS polymers, embedded with Pt nanoparticles	0.15	0.3% H_2_O_2_	23	153.3	Self‐assembling, dialysis	[98]
	Hollow sphere	One hemisphere: SiO_2_ shell with Pt inner layer, The other: mSiO_2_ shells	0.5 diffusiophoresis	10% H_2_O_2_	1.49b)	NAa)	Sol–gel technique, selective etching	[99]
			1.5 electrophoresis		14	9.3		
			3 diffusio + electro‐phoresis		NAa)	NAa)		
		Pt@SiO_2_/Au	0.495	3% H_2_O_2_	NAa)	NAa)	Ion‐ligand interaction, sputtering	[103]
	Solid sphere	PNIPAM @Au/Pt	2	1.5% H_2_O_2_	8.5@25C°	4.25	Sputtering, grafting	[101]
					2.3@35C°	1.15		
		TiO_2_/Pt	1.2	2.5% H_2_O_2_	7.1 with UV off	5.92	Sputtering	[104]
					13.8 with UV on	11.5		
		TiO_2_/Au	3.5	12% H_2_O_2_	18 under UV light	5.14	Sputtering	[106]
					5 under green light	1.43		
	Core/ shell rod	Au/Ru bimetal	3.5	5% H_2_O_2_	30.1	8.6	Template‐assisted electrodeposition	[100]
	Pot‐like microstructure	Ureases @SiO_2_/Au	2.3	0.6 mm urea	6.2	2.70	Template‐etching method,	[102]
				50 mm urea	18	7.83	Bioconjugate technique	
	Brush‐shaped nanorod	Zn/Pt	2	3% H_2_O_2_	7.5 with UV off	3.75	Etching, electron‐beam evaporation	[105]
					29 with UV on	14.5		

a) NA = not available;

b) the entry denotes the diffusion coefficient (μm s^−2^).

## Conflict of Interest

The authors declare no conflict of interest.
